# Elucidating Sex-Specific Immune Profiles in a Breast Cancer Model

**DOI:** 10.3390/ijms252313113

**Published:** 2024-12-06

**Authors:** Ebony Hargrove-Wiley, Dora Obodo, Wendy Bindeman, Barbara Fingleton

**Affiliations:** 1 Program in Cancer Biology, Department of Pharmacology, Vanderbilt University, Nashville, TN 37232, USA; ebony.l.hargrove-wiley@vanderbilt.edu (E.H.-W.); wendy.e.bindeman@vanderbilt.edu (W.B.); 2 Department of Biostatistics, St. Jude Children’s Research Hospital, Memphis, TN 38105, USA; dora.obodo@stjude.org

**Keywords:** sex differences, breast cancer, male breast cancer, tumor immunology

## Abstract

Breast cancer is commonly thought of as a “women’s disease”. However, men are increasingly diagnosed with the disease, and their mortality rates are disparately higher than those of female patients. The abundance and composition of the immune microenvironment are determinants of breast cancer progression and survival. It is well documented that there are sex-specific differences in the immune response to several diseases, including various cancers. However, the effects of these differences in the context of breast cancer remain to be explored. This study demonstrates sex differences in the hormonal and immune landscape of the MMTV-PyMT transgenic murine model of female and male ER+ breast cancer using single-cell RNA sequencing (scRNA-Seq), whole-slide immunohistochemistry, and flow cytometry. Mammary tumors of transgenic male mice had increased estrogen receptor alpha expression and enriched nuclear binding signatures compared to female tumors. In the tumor immune compartment, male mice had lower intratumoral leukocyte infiltration. Yet, scRNA-Seq analysis reveals a more immunostimulatory microenvironment and increased antitumor immune populations in the primary and metastatic lungs as compared to transgenic females. Despite a more favorable innate immune profile, the metastatic burden was increased in male mice. Our data support a sex-dependent immune response in mammary carcinoma associated with the tumor, and likely host, hormonal environment. With emerging therapeutics targeting the tumor immune microenvironment, characterizing immune profiles is critical for optimizing their use in all breast cancer patients.

## 1. Introduction

Male breast cancer is a rare but increasingly prevalent medical condition that often remains overlooked in discussions about breast health [1,2]. Male and female breast cancer share common risk factors, clinical presentation, and treatment approaches. However, clear sex disparities exist concerning breast cancer progression and survival outcomes that are only partly explained by the older age and stage of diagnosis in male patients [3,4,5,6,7]. The gender-associated breast cancers are molecularly and biologically distinct diseases. Yet, our knowledge of male disease biology is limited due to the lack of male inclusion in preclinical and clinical investigations [8,9]. A greater understanding of male breast cancer biology is vital for effective management of the disease.

The immune system is pivotal in combating cancer [10]. There is substantial evidence describing the differences between the immune responses of males and females, ranging from susceptibility to certain diseases to the efficacy of treatments [11,12,13,14,15]. While social and environmental aspects influence these differences, biological and hormonal factors directly regulate the maturation and function of immune populations [16,17,18]. Hormonal factors are also key determinants in certain breast cancer subtypes. In terms of breast cancer, estrogen-receptor-driven Luminal A and B subtypes are most prevalent in both female and male patients. In this context, estrogen receptor (ER) abundance and stimulation promote tumor development and the suppression of immune cells [19,20,21]. The androgen receptor (AR) is highly expressed in these subtypes as well and is shown to impact tumor growth and decrease immune infiltration [22,23,24]. In other malignancies, androgens are shown to inhibit immune cell effector functions [25,26,27]. However, the effects of androgen on the specific immune populations in the breast tumor immune microenvironment are poorly studied [28].

Understanding these sex-specific nuances in the cancer-immune interaction associated with sex is crucial for tailoring therapeutic approaches. This study examined the immune compartments of multiple organs involved in mammary tumorigenesis and metastatic expansion in female and male MMTV-PyMT transgenic mice. We confirmed that this model resembled human Luminal B breast cancer, as previously reported. Similar to the events in human breast cancer, male mice experienced delayed tumor initiation and a greater degree of pulmonary metastasis. Female and male primary tumors had diverse transcriptomic profiles. Male tumors were more prominently enriched for pathways that support tumor expansion and immune mediation. Immune populations derived from male primary tumors and metastatic lungs had larger proportions of Cd11b+ myeloid cells and MHC2+ antigen-presenting cells than female primary tumors. Conversely, splenic neutrophils were elevated in female mice as compared to males. Furthermore, the quantification of plasma proteins identified CC motif chemokine ligand 6 (CCL6) as a potential sex-specific mediator of immune signaling. Viewed collectively, our findings illuminate sex-specific differences in the mammary tumor immune microenvironment and identify tumor intrinsic gene pathways that likely support variances in disease progression and anticancer immunity.

## 2. Results

### 2.1. Disease Progression and Classification of PyMT-Induced Female and Male Mammary Tumors

**Figure 1:**
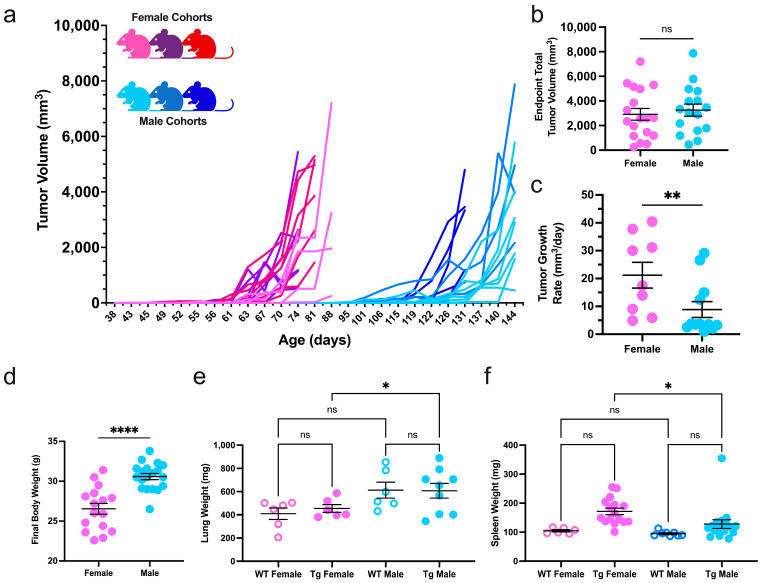
Disease kinetics in female and male mammary carcinoma. (**a**) Caliper measurements of total tumor burden for three cohorts of transgenic female (shades of pink) and male (shades of blue) MMTV-PyMT mice; (**b**) total tumor volume at the study endpoint (females = 18, males = 16) and (**c**) the growth rate of the first palpable tumor of each mouse were compared between the sexes (females = 9, males = 12); (**d**) the final body, lung, and spleen weights of MMTV-PyMT (Tg) mice compared to non-transgenic littermates (WT); (**e**) spleen and (**f**) lung weights in non-tumor- and tumor-bearing female and male cohorts. All metrics were compared with a Student’s *t*-test or two-way ANOVA where applicable. * *p* ≤ 0.05, ** *p* ≤ 0.01, **** *p* ≤ 0.0001, and ns = nonsignificant.

The MMTV-PyMT transgene induces multifocal mammary carcinoma in FVB/n mice at a high penetrance in both sexes [29,30]. PyMT mice from three distinct generations underwent bi-weekly body assessments to detect mammary tumorigenesis and monitor disease progression and overall well-being. As previously established [30,31], transgenic females developed palpable tumors earlier than males (61.78 ± 8 and 118.1 ± 12 days old, respectively, Figure 1a and Figure S1a). On average, transgenic female mice developed more palpable primary mammary tumors as compared to males (Figure S1b). Multifocal tumors were measured to calculate total tumor volume throughout the study. At the study endpoint, female and male mice had similar total tumor volumes. However, transgenic males had a slower tumor growth rate (Figure 1b,c). Unsurprisingly, transgenic male mice had a higher final body weight and lung mass than female mice (Figure 1d,e). Additionally, females had significantly enlarged spleens compared to male counterparts, indicating systemic immune hyperactivity induced in tumor-bearing female mice (Figure 1f).

**Figure 2:**
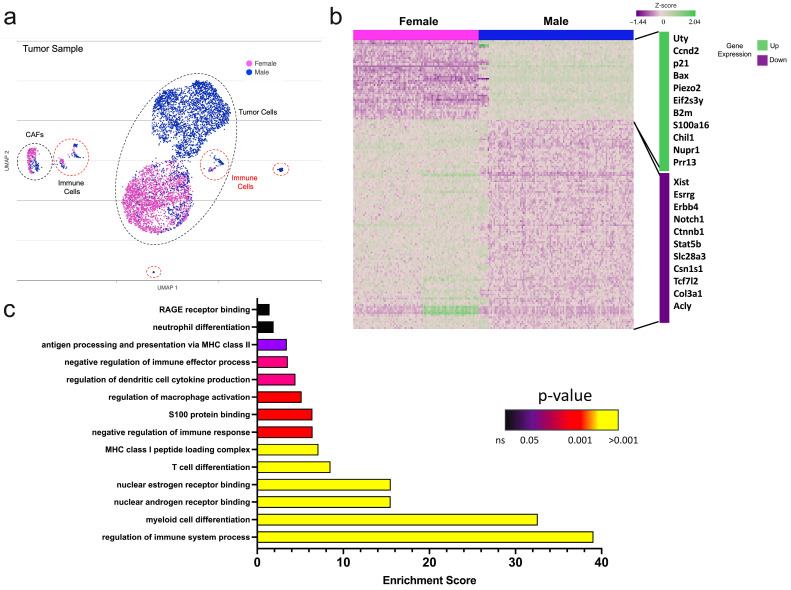
Male tumors are enriched for immunomodulatory genes. (**a**–**c**) Single-cell RNA sequencing of female and male PyMT tumors. (**a**) UMAP of the tumor samples using Partek Flow; (**b**) heatmap of differentially expressed genes. The list highlights sex-associated and immune-related genes that were amongst the most differentially expressed in male versus female tumors. (**c**) Gene set enrichment analysis of immune-related biological processes in male versus female tumors.

Next, single-cell RNA sequencing (scRNAseq) was performed on whole tumor samples to determine the gene expression profile of tumors from each sex. Tumor cells were confirmed through the expression of various epithelial-specific transcripts such as cytokeratin and E-cadherin and the lack of immune and stroma-related genes. Female and male tumor samples had considerable heterogeneity (Figure 2a,b). Differential analysis of tumors demonstrated that male tumors had greater transcript expression of cell cycle regulators p21 and Ccnd2, and the antiapoptotic gene Bax. These genes contributed to the overall enrichment of gene sets associated with the positive regulation of cell growth observed in male tumors. Genes previously linked to metastasis, Bcl2, and Notch were downregulated in male samples compared to females; however, broad pathways involved in cell migration mediation, such as the advanced glycation end products (AGE), S100 binding proteins, and their cognate receptor (RAGE) signaling cascades (Figure 2c and Figure S1e) were enriched. These data highlight a complex gene regulatory network based on sex and likely support sex differences in mammary tumor development and spread.

Additionally, the molecular subtype of tumors was characterized using scRNAseq. The tumor cells from both sexes had similar transcript levels of estrogen receptor alpha (Esr1), androgen receptor (Ar), and human epidermal growth factor receptors (Erbb2, Erbb3), which are the attributes of the Luminal B breast cancer subtype. Male tumors expressed lower levels of Erbb4 (Figure S1c), a gene implicated in aggressive breast cancer phenotypes [32]. Progesterone receptor was not detected in tumors from either sex. Gene ontology assessment revealed enrichment for estrogen and androgen nuclear receptor binding in male tumors, suggesting increased hormonal regulation in male mice (Figure 2c). These findings confirm that PyMT-driven murine tumors molecularly and cellularly resemble AR-positive Luminal B breast cancer and have effective hormone receptor gene regulation likely because of extracellular hormonal stimuli.

### 2.2. Sex Determines the Tumor Immune Microenvironment

**Figure 3:**
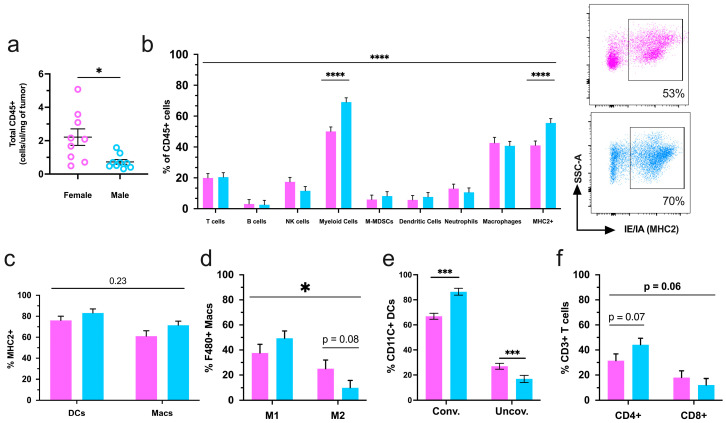
Intratumoral immune infiltration is decreased in male tumors however, male mice have a greater representation of tumor-suppressing immune populations. (**a**) Normalized totals of CD45+ immune cells in transgenic female and male mammary tumors; (**b**) flow cytometry analysis of the intratumoral immune cell composition in MMTV-PyMT mice with representative dot plots of MHC2+ populations from both sexes; (**c**) the percentage of MHC class II (MHC2) dendritic cells (DCs) and macrophages (macs); (**d**) parent of parent subsets of M1 (Cd11b+, CD206-) and M2 (Cd11b+, CD206+) macrophages; (**e**) conventional (CD11b+) and unconventional (CD11b-) dendritic cells and (**f**) T-cell populations. Graphs depict the pooled data from three cohorts of female (*n* = 16) and male (*n* = 16) mice. Natural killer (NK), monocytic-myeloid-derived suppressor cells (M-mdscs). Female mice are represented in pink, and males are represented in blue for the entire figure. * *p* ≤ 0.05, *** *p* ≤ 0.001, and **** *p* ≤ 0.0001.

We then used flow cytometry to comprehensively interrogate the primary tumor immune microenvironment in male versus female mice. Although there were no sex differences in total tumor volume at the end of the study, flow cytometry analysis of tumors determined that males had decreased CD45+ immune infiltration as compared to females (Figure 3a). The composition of the major immune cell populations (%CD45+) was assessed to include: T-cells (Cd3+), B-cells (B220+), natural killer cells (NK1.1), pan-myeloid cells (Cd11b+), monocytic-myeloid-derived-suppressor cells (M-MDSC, Cd11b+, MHC2-, Ly6G-), dendritic cells (Cd11c+), neutrophils (Ly6G+), macrophages (F480+), and immune cells positive for major histocompatibility complex class II (MHC2+). Among these populations, T-cells, natural killer, and myeloid cells, specifically macrophages, were the most prominent within mammary tumors from both sexes.

Cd11b+ myeloid cells were in higher proportions in male tumors, and the myeloid lineage cell types M-MDSC, DC, neutrophil, and macrophage showed no differences between the sexes. Moreover, MHC2-expressing cells were elevated in the immune compartment of male tumors (Figure 3b). Macrophages and dendritic cells (DC) are antigen-presenting cells (APCs) that upregulate MHC2 upon successful activation [33,34]. In males, the overall increase in MHC2+ cells was supported by a greater percentage of MHC2+ in these two APC populations (Figure 3c). Another indicator of macrophage function is the presence of the CD206 surface marker, also known as the mannose receptor [35], which is expressed on tumor-promoting M2-like macrophages. Intratumoral percentages of tumor-suppressing M1-like macrophages (Cd11b+, CD206-) were elevated in male mice, while M2-like (Cd11b+, CD206+) macrophages were increased in females (Figure 3d). In addition, DCs exhibit antitumor phenotypes through conventional activation and the sustained expression of myeloid cell marker Cd11b. Conversely, Cd11b-negative DCs are shown to have unconventional activation and support tumor growth [33]. Male tumors had increased conventional and decreased unconventional DCs compared to tumors from female mice (Figure 3e). Lastly, mammary tumors displayed modest, albeit nonsignificant, sex-associated differences in the ratios of T-helper (CD4+) and cytotoxic (CD8+) T-lymphocyte subsets (Figure 3f).

Additional pathway enrichment analysis of sex-specific PyMT tumors revealed differences in immune signaling. Compared to female tumors, male tumors were highly enriched for genes associated with antigen presentation and myeloid and lymphocyte recruitment and activation. In concordance with these immunostimulatory pathways, male tumors were moderately enhanced for genes involved in PD-L1 expression, suggesting differential tumor-immune interactions based on sex (Figure 2c and Figure S1d, Table S1).

**Figure 4:**
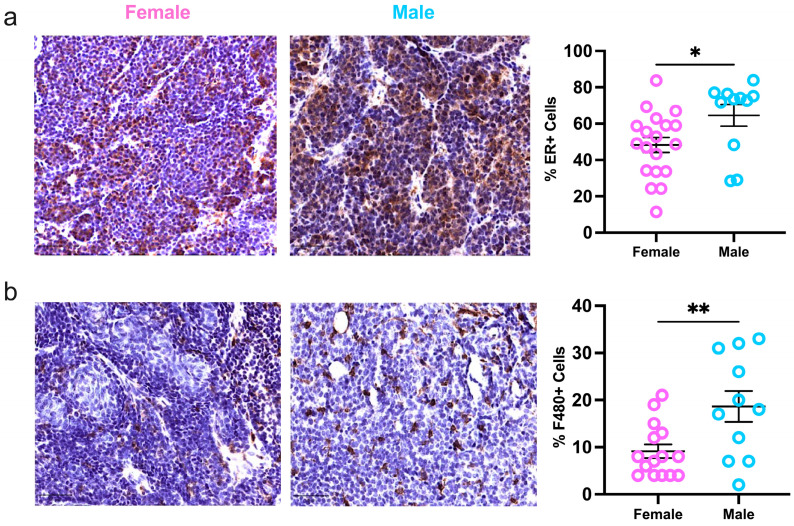

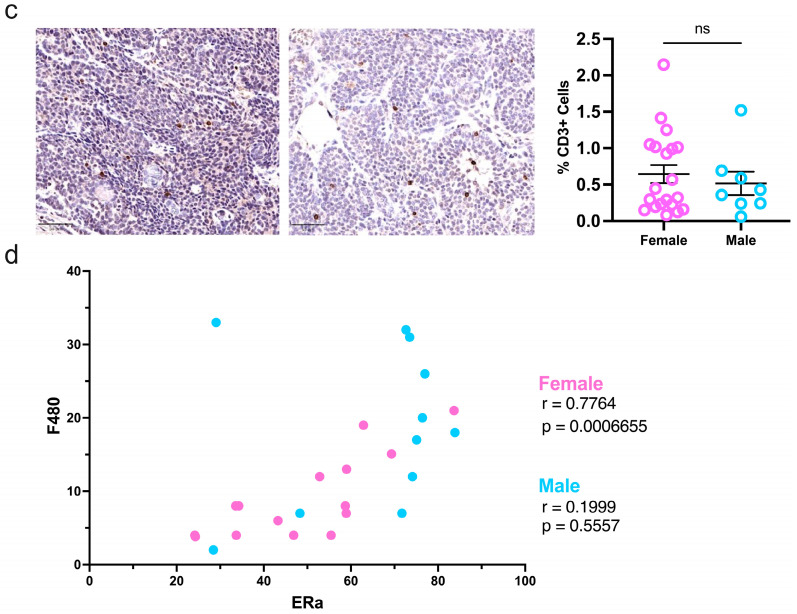
Estrogen receptor expression and macrophage infiltration are heightened but not correlated in male tumors. Quantification of (**a**) ERα, (**b**) F480-positive macrophages, and (**c**) CD3-positive T-cells in whole slide mammary tumors; (**d**) correlation of ERa and F480 intratumoral protein expression. Metrics were assessed with a student’s parametric or nonparametric test or Pearson’s correlation analysis where applicable. Bars represent 50 μm; * *p* ≤ 0.05, ** *p* ≤ 0.01, and ns = nonsignificant.

Finally, due to increased hormonal signaling in male tumors reported by the scRNAseq data, immunohistochemistry was used to evaluate ERα protein expression in sex-specific MMTV-PYMT tumors. In male mice, more tumor cells (65%) were positive for ERα compared to female tumors (48%; Figure 4a). Immune cell infiltration and activation are important for regulating cancer progression. However, the immune profile of luminal cancers, especially in males, is poorly understood [10]. To study the immune compartment, consecutive tumor sections were stained for F480+ macrophages and CD3+ T-cells. Aligned with the flow cytometry data, macrophages were highly represented in mammary tumors of both sexes. Male tumors demonstrated increased macrophage infiltration compared to females, and no sex differences were reflected in the presence of T-cells (Figure 4b,c). Moreover, female but not male tumors had a positive correlation between macrophage infiltration and ERα expression (Figure 4d). Estrogen is shown to recruit tumor-associated macrophages [36]. However, these data suggest the estrogen receptor status of mammary tumors significantly influences the immune response of females and is less important in males despite its increased presence in male tumors. Taken together, these data support a divergence of intratumoral immune regulation and activation between female and male tumor-bearing MMTV-PyMT mice.

### 2.3. Tumor Burden Creates a Distinctive Pulmonary Immune Composition as a Function of Sex

**Figure 5:**
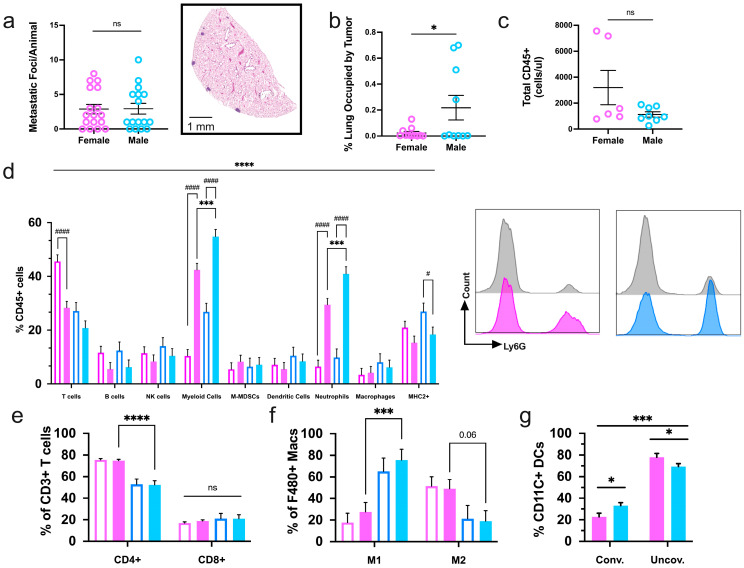
Mammary carcinoma progression induces sex-specific immune profiles in the whole lungs of PyMT mice. (**a**) The sum of metastatic foci from three planes of lung tissue harvested from tumor-bearing MMTV-PyMT female and male mice. An H&E image of a plane of lung tissue with four visible metastatic foci (**right**); (**b**) the area of metastatic foci compared to the total lung area. (**c**) Quantification of total immune cells (CD45+) and (**d**) the proportions of subpopulations were analyzed by flow cytometry in lungs from tumor-bearing female tumor-bearing female (pink) and male (blue) MMTV-PyMT mice. Transgenic mice (solid bars) were assessed alongside sex- and age-matched non-transgenic littermates (unfilled bars). Neutrophils (CD45+, Ly6G+) proportions from transgenic female (pink) and male (blue) mice and sex- and age-matched non-transgenic littermates (grey) are represented by histograms (**right**); (**e**) subsets of T-cells, (**f**) macrophages, and (**g**) dendritic cell populations. Graphs depict the pooled data from two cohorts of transgenic and non-transgenic female and male mice (*n* ≥ 6). * or ^#^ *p* ≤ 0.05, *** *p* ≤ 0.001, **** or ^####^ *p* ≤ 0.0001 and ns = nonsignificant.

PyMT-induced mammary tumors primarily migrate to the lungs to produce micrometastases [37,38]. On average, female and male mice developed a comparable number of metastatic foci, 4.4 and 2.9, respectively (Figure 5a). However, transgenic males had a more severe overall lung tumor burden than females, as determined by histological assessment (Figure 5b). On the other hand, absolute immune infiltration did not vary at the secondary site (Figure 5c). Mammary tumor progression did result in a unique pulmonary immune compartment that was largely compromised of T-cells, neutrophils, and equal parts B-cells and other myeloid cell types. In comparison to sex-matched non-transgenic littermates that do not develop tumors, transgenic mice had a greater percentage of myeloid cells, largely of the neutrophil subclass, in mice of both sexes. Additionally, transgenic females had significantly decreased proportions of T-cells in the lung, whereas transgenic males had reduced MHC2+ cells and revealed similar MHC2+ ratios as non-transgenic mice of the same sex (Figure 5d). Comparable, though nonsignificant, differences in the pulmonary immune profile were seen between transgenic and non-transgenic mice of the opposite sex. The immune profile within the lungs of transgenic mice exhibits a shift from being lymphocyte- to myeloid cell-dominant compared to sex- and age-matched non-transgenic littermates.

Immunophenotyping of transgenic mouse lungs further supported a differing immune profile based on sex. Interestingly, CD4+ T-helper cells were diminished in male mice, but no differences were seen in CD8+ cytotoxic T-cell levels (Figure 5e). As in the primary mammary tumor immune microenvironment, total myeloid cells were elevated in the lungs of males, although in this case, it was largely due to increased neutrophil counts (Figure 5e). Tumor-opposing M1-like macrophages and conventional DCs were also enriched in male lungs compared to females (Figure 5f,g). In all, male mice present an apparently more favorable anti-tumor immune composition in their lungs in response to mammary carcinoma spread.

### 2.4. Male and Female Mice Have Unique Systemic Immunobiology

**Figure 6:**
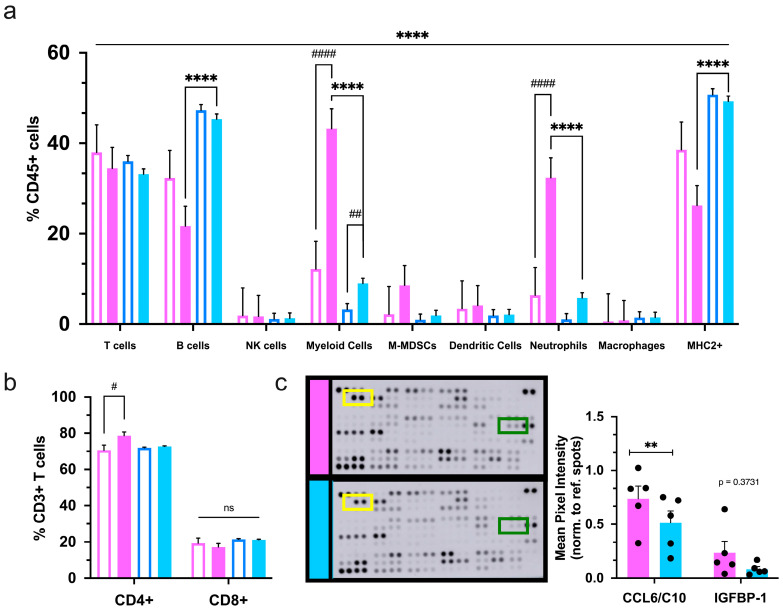
Systemic immune composition and signaling varies based on mammary tumor presence and sex. (**a**) The frequency of immune populations was analyzed by flow cytometry in the spleens of non-transgenic (unfilled bars) and tumor-bearing transgenic (solid bars) female (pink) and male (blue) mice; (**b**) assessment of T-cell subsets in mouse spleens. Graphs depict the pooled data from three separate cohorts of transgenic and non-transgenic female and male mice (*n* ≥ 16); (**c**) immune-related proteins were quantified from the plasma of transgenic mice at the end of the study. Shown are representative immunoblots of multiprotein array from transgenic mice of each sex (**left**) with quantitation of changes on the right, (*n* = 5 per sex). The yellow and green boxes on the blots highlight CCL6 and IGFBP-1, respectively. Significance was determined by two-way ANOVA. ^#^ *p* ≤ 0.05, ** or ^##^ *p* ≤ 0.01, **** or ^####^ *p* ≤ 0.0001 and ns = nonsignificant.

Flow cytometry analysis of the spleen was performed to evaluate the immune response at the systemic level. As in healthy mice, T- and B-lymphocytes constituted most splenic immune cells. Males and females have a more prominent B-cell and T-cell population, respectively, than the opposite sex at baseline. These phenotypes were confirmed in non-tumor-bearing FVB/N mice of varying ages (Figure S2a–c). In MMTV-PyMT mice, elevated B-cell ratios were conserved in males; however, female spleens no longer displayed an increased T-cell population compared to males (Figure 6a). Interestingly, transgenic, tumor-bearing females had a relatively lower proportion of B-cells than neutrophils, which is not seen in normal physiology. The neutrophil population drastically increased in tumor-bearing females compared to tumor-bearing males and both tumor-naive female and male mice. (Figure 6a). More specifically, this phenomenon caused transgenic females to have an enrichment of total myeloid cells in the spleen, which differs from the situation within the primary tumors and lungs. Transgenic females displayed a rise in T-helper cells as compared to non-tumor-bearing mice. Cytotoxic T-cells showed no differences between non-transgenic and transgenic mice of either sex (Figure 6b).

Lastly, quantification of 111 immune signaling proteins from the plasma of transgenic mice revealed that females had higher levels of chemokine CCL6 (C10), a principal chemokine for monocytes, macrophages, neutrophils, and T-cell recruitment (Figure 6c). Taken together, these data indicate that female and male mice had varying mechanisms of immune signaling that may exacerbate or diminish when challenged with mammary carcinoma and thus may differentially impact the trafficking and regulation of immune cells associated with this disease.

## 3. Discussion

Many recent studies emphasize the importance of sex as a biological determinant of antitumor immunity [13,25,39]. In this study, we employed a murine model of breast cancer to comprehensively define the immune response to mammary cancer based on the sex of the animal. Our transcriptomic and histologic findings indicate that PyMT-driven tumors are estrogen-, androgen-, and HER2-positive, recapitulating the Luminal B subtype in humans. We demonstrate that male and female tumors had distinct levels of HR expression, gene signatures, and pathways related to proliferation, cell survival, and immune modulation. Immunophenotypic analysis of PyMT tumors revealed increased overall immune cell infiltration in female tumors. Yet, the major immune populations remained unchanged between the sexes, with macrophages, T lymphocytes, and NK cells being most highly represented. Comparatively, males exhibited greater proportions of Cd11b+ myeloid, MHC2+, and tumor-suppressing macrophage and dendritic cells in primary tumors with overlapping differences in metastatic lungs. Contrastingly, myeloid-lineage neutrophils were elevated in transgenic female spleens compared to males. Although male mammary tumors lack significant immune cell infiltration, they are more immune activating and promote a phenotypically favorable innate immune profile.

Polyoma middle T-antigen is a powerful mouse oncogene that activates the Src/Akt pathway, which is highly relevant in several human malignancies, including breast cancer [30]. In the early phases of tumor development, mammary cells induced by PyMT express luminal markers and respond to hormone stimulation [40,41]. In later stages, loss of ER has been demonstrated [42,43], although AR is maintained in lung metastases [43]. Thus, the tumoral and global hormonal environment influence the biology of PyMT tumors. We demonstrate that female mice had a shorter tumor latency and expansion than male mice. This observation aligns with the disease kinetics seen in human breast cancer patients [7,44] and is, in part, due to ER+ tumor cell dependency upon estrogen and/or the earlier maturation of mammary tissue and, thus, the expression of the transgene promoter MMTV in female mice [45]. It is well established that ER and AR signaling induces cell growth in certain breast cancer subtypes [28,46].

Interestingly, male tumors displayed increased ER-positivity by IHC and nuclear receptor binding per gene set enrichment analysis, likely directly associated with receptor expression. However, we did not directly measure DNA binding activity and cannot be sure of the nuclear receptor activity. Overall, the data support that variances in the tumoral hormonal status and downstream signaling could differentially alter tumor growth potential and components of its microenvironment [19,20,21]. Single-cell transcriptomics showed that male tumors had increases in additional pathways for proliferation and metastasis, demonstrating a more aggressive disease partially mediated by endocrine receptor growth signals. Male tumors had higher levels of proinflammatory pathways including specific transcripts, *S100a16* and *Piezo2* [47,48]. These inflammatory signals can also support proliferative activity and metastatic spread. Our data suggest that increased immune-related signals may be associated with stemness and proliferation as demonstrated by the upregulation of genes like *p21*, *Ccnd2*, and *Notch* and further mediated by increased ER levels. Additionally, apoptosis-pathway genes, *Bax* and *Bcl2,* are also overexpressed in male tumors. These data, along with the slower growth rate of the male tumors and increased proportions of tumor-suppressing M1-like macrophages and conventional DCS, suggest that males have effective antitumor immunity; however, this may also drive metastatic spread.

Male tumors showed relatively reduced immune infiltration; thus, upregulation of immunostimulatory genes may serve as a compensation mechanism to boost the immune response. Similar observations were seen in resected tumors from male lung adenocarcinoma patients [49] and in other cancers where male tumors demonstrated increased tumor mutational burden, which supported enhanced tumor antigenicity compared to female tumors [50]. Although hormonal and immune signatures were more evident in male tumors, histochemical analysis of ER, pan-macrophages, and T-cells did not support a correlation between the hormone receptor and these immune populations at the protein level. Contrastingly, female tumors exhibited a significant association between ERα expression and intratumoral macrophages. Global administration and stimulation of this hormone receptor have been linked to macrophage recruitment in several reports [36]. However, these studies did not include male models. Our data suggest estrogen influences the immune landscape differently in female and male mammary tumors. Thus, targeted hormone therapies, like tamoxifen, may be less effective in reversing estrogen-induced immunosuppression in male patients. Instead, alternative treatments like androgen receptor blockade, stimulation, or immunotherapy should be considered and have been suggested for breast cancer and AR-responsive prostate cancer patients [24,51,52,53]. However, a closer assessment of the AR/ER protein ratios in ER+ breast cancer is crucial, given its dual effects on tumor cell growth and immunosuppressive effects.

We report that transgenic male mice had elevated proportions of Cd11b+ myeloid cells and, therefore, an increased myeloid-to-lymphocyte ratio compared to females. In the context of cancer, a higher myeloid cell intratumoral ratio reflects an immunosuppressive microenvironment and is associated with a worse prognosis in several cancers [54,55,56]. Intriguingly, M1-like macrophages and conventionally activated DCs make up a larger portion of the male immune compartment; however, this increase in antitumor immune cells was insufficient to diminish tumor growth. The inability to control tumor growth is likely due to the lower abundance of infiltrated cells and is worsened by deficiencies in innate cell function or the crosstalk to adaptive effector cells. We discovered no differences in total CD4+ or CD8+ T-cell percentages within mammary tumors. Based on other reports, male-associated androgens preferentially differentiate cytotoxic CD8+ to more exhausted phenotypes and render T-cells unresponsive to antigen presentation and stimulation [25,26,27]. Moreover, male tumors had greater activation of PD-L1 expression pathways, which could further impede infiltrated immune cells’ phagocytosis and cytotoxic functions [57].

Male cancer patients have higher rates of metastases that likely contribute to lower survival rates [4,5]. Although our model showed a similar number of micrometastases in the lungs of both sexes, we observed an increase in the size of spontaneous lung-metastatic foci in male mice. These data correlated with enriched cellular migration pathways reported in male primary tumors. Generally, the lungs have a robust immune compartment. Tumor-bearing female and male mice had comparable levels of pulmonary immune infiltration. However, mammary cancer progression induced several changes in the lung immune profile. Female and male transgenic mice presented high percentages of neutrophils in the lungs, with transgenic males surpassing the levels detected in females. Many cancers give rise to high neutrophil levels, which can support tumor progression. In liver metastases, neutrophils aid in tumor cell spread in an androgen receptor-dependent manner [58]. Given that PyMT lung metastases retain AR expression [43], the hormonal environment of male mice likely mediates neutrophil accumulation in the lungs that supports accelerated secondary tumor propagation [59,60].

T-cells were reduced in female mice, indicating a weakened adaptive immune response. On the other hand, CD4+ T-helper and MHC2+ cells were decreased in male mice, suggesting reduced immune activation and, thus, inefficient tumor surveillance, which encourages secondary colonization. Similarly to primary tumors, myeloid cell proportions were increased in the lungs of male mice alongside M1-like macrophages and canonical DCs. Although this would suggest more antitumorigenic activity, the lower levels of CD4+ T-helper cells could hinder immunosurveillance in male tumors. T-helper cells are essential for direct tumor cell killing and CD8+ T cell effector functions [61,62,63]. The reduced ability to control lung tumor dissemination could result from lower proportions of T-helper cells. Conversely, females had increased M2-like macrophages, which can induce exhaustive T-cell phenotypes [64,65]. These variations suggest that each sex has differential mechanisms to suppress tumor immunity that further promotes disease progression.

We lastly determined the systemic immune profile of transgenic animals by assessing the spleen and compared this with the systemic profile in matched non-transgenic controls. Male and female spleens from tumor-bearing mice had abnormally high proportions of Cd11b+ myeloid cells, specifically neutrophils, compared to their age- and sex-matched non-tumor-bearing littermates. In contrast to what we observed in the metastatic lungs, transgenic females, not males, showed a more prominent rise in their neutrophil population in the spleen, and this neutrophil outgrowth likely caused the relative decrease of B-cells in transgenic females. As mentioned above, neutrophil levels can reflect the magnitude of the disease [66]. Accordingly, increased neutrophils in the spleen but not the lungs are likely in response to the increased development of primary tumors in transgenic females and not due to the advancement of secondary tumors in the spleen or other organs [37,67]. However, the presence of secondary tumor cells in organs other than the lungs was not examined. Furthermore, increased systemic CCL6, defined as a chemoattractant for neutrophils, could contribute to the elevated levels of this immune population [60]. In all, these distinctions denote sex- and organ-specific immune cell recruitment and trafficking influenced by immune signaling at the primary and secondary organ sites.

This study has potential limitations. We associate immune profiles based on sex; however, sex is not a causal mechanism [68]. To investigate the role of sex hormones in shaping these profiles, we histologically validated and quantified the expression level of the estrogen receptor within tumors and presented evidence of receptor binding in our single-cell sequencing analysis. However, due to difficulties with reagents, we did not evaluate AR protein levels or localization, nor did we measure intratumoral or serum levels of estrogens and androgens. Thus, we cannot confidently state that AR is present in the nucleus of tumor cells, or that the ER that is present is responding to any level of estrogen in the system. It is well established that human and murine post-pubertal estrogen and androgen levels are higher in females and males, respectively. The non-transgenic and transgenic mice used in this study were of post-pubertal age (older than two months) and younger than 18 months, the age at which mice are considered old and associated with hormonal decline. The bioavailability of sex hormone levels does not change significantly within this age range, and they are likely accounted for at their variable endogenous levels in our male and female mice cohorts [69]. Additionally, several studies have compared age-related immunological differences. Unfortunately, these reports focus on two- to four-month-old and 17- to 24-month-old mice, or the human equivalent age. Those studies do not include a comparison of five- to six-month-old mice like the age of males in our study or of FVB genetic background [18,70,71,72,73]. Beheshti and colleagues reported that mammary tumor-bearing adolescent and young adult C57 mice had highly similar immune-related transcriptional profiles but did not assess the influence of sex in these investigations [74]. Through flow cytometry immunophenotyping of the spleen, we identified very modest changes in T-cells related to sex and age (two- versus five-month-old, Figure S2b,c). Based on these reports and data, we believe that our data mostly reflect sex differences induced by mammary carcinogenesis and not from age.

Furthermore, to prepare whole tumors for scRNAseq analysis, samples underwent enzymatic disassociation and dead cell removal, which can stress cells and alter their transcriptional profile [75]. Moreover, immune and other stromal cells were not sufficiently extracted with our chosen sequencing method; therefore, transcriptomic interpretations reflect data from tumor and stromal populations.

Numerous studies have emphasized sex biases in antitumor immunity [50,76]. Estrogens and androgens are largely responsible for sex disparities in the immune response to many diseases, including various non-reproductive cancers [27,28,77]. We broadly describe the hormonal status and sex-dependent immune response in a model of female and male ER+ breast cancer for the first time. Our findings elucidate variances in hormone receptor expression, immune signaling pathway stimulation, and distinctive immune microenvironments based on sex. These observations suggest that female and male breast cancer are biologically different based on their hormonal environments and their effects on the immune system. These data endorse more rigorous translational and preclinical male breast cancer biology investigations. Furthermore, this study warrants the inclusion of male patients in clinical research to better understand and identify the implications of immunotherapeutic treatment options in these patients.

## 4. Materials and Methods

### 4.1. MMTV-PyMT Murine Model of Breast Cancer

Male MMTV-PyMT mice [FVB/N-Tg(MMTV-PyVT)643 Mul/J] [29] were originally obtained from Jackson Labs (Bar Harbor, ME, USA) and used to establish an in-house breeding colony. Transgenic genotyping was performed by Transnetyx (Memphis, TN, USA) using real-time PCR. Transgenic mice were assessed twice weekly for overall health and the detection and measurement of tumors by manual caliper. Tumor volume was calculated with the equation 0.5 (width^2^ × length), and each cohort was euthanized when one animal within it reached the humane endpoint. Total tumor burden is the volume of all palpable tumors at the measurement time. The growth rate of tumors was determined by computing the linear slope of individual growth curves. Only tumors with linear phases that lasted for 3 or more timepoints were included in the analysis. All experimental animal protocols were conducted only after review and approval by the local institutional animal care and use committee.

### 4.2. Single-Cell Sequencing of Sex-Specific Tumor Cells

Mammary tumors were excised and disassociated, as reported previously [53]. The EasySep dead-cell removal kit (Cat. No. 17899, StemCell Technologies, Vancouver, BC, Canada) was used to improve the viability quality of the sample for subsequent processing. For sequencing, 50,000 tumor cells were lysed, and cDNA was prepared using the PIPseq™ T20 3’ Single Cell RNA Kit v4.0 (Fluent BioSciences, Watertown, MA, USA) following the manufacturer’s protocol [78]. Single-cell sequencing was performed on a NovaSeq 6000 (Illumina Inc,. San Diego, CA, USA) by the Vanderbilt Technologies for Advanced Genomics (VANTAGE) core. Data were processed in PIP Seeker previously detailed [78]. Gene-specific and pathway analysis was performed using Partek™ Flow™ Explore Spatial Multiomics Data using Partek™ Flow™ software, v11.0.

### 4.3. Histological Analysis of the Breast Tumor Murine Model

Standard immunohistochemistry (IHC) was performed on male and female mammary tumors. In brief, tissues were collected and fixed overnight in 10% buffered formalin, dehydrated in ethanol, and embedded in paraffin. Following deparaffinization, slides underwent endogenous peroxidase blocking followed by heat-induced antigen retrieval. Whole primary tumors were stained for ERα (4 µg/mL; Cat. No. MBS2027687 MyBioSource, San Diego, CA, USA), CD3 (0.5 µg/mL; Cat. No. SC-1127, Santa Cruz Biotechnology, Dallas, TX, USA), and F480 (1.5 µg/mL; Cat. No. 70076S, Cell Signaling Technology, Danvers, MA, USA). The appropriate positive and negative control tissues were included in the experimentation. Negative controls were incubated with IgG in 10% goat or rabbit serum in tris-buffered saline (TBS), depending on the secondary antibody used. Whole slide imaging was performed in the Digital Histology Shared Resource at Vanderbilt University Medical Center at 20× magnification. Positive staining was quantified using the Qupath positive cell detection automated function [79].

Lungs were inflated with cold phosphate-buffered saline (PBS) and processed as described above. FFPE lungs were sectioned at three tissue depths 200 μm apart to ensure an evaluation of the tissue in different regions. Lungs were stained with hematoxylin and eosin (H&E), and images were captured using the Zeiss Axioscan whole slide scanner (Zeiss Microscopy, Jena, Germany). The number of metastatic foci and metastatic area (area of tumor: area of normal lung tissue) was assessed using QuPath v0.5.1 [79].

### 4.4. Immunophenotyping of the Immune Microenvironment

Mammary tumors, lungs, and spleens were harvested, as previously mentioned above. For flow cytometry, one million cells were incubated with Fc block for 20 min, followed by a 30 min incubation at 4C with 7-AAD viability dye and fluorophore-conjugated primary antibodies purchased from BioLegend (San Diego, CA, USA): CD45-APC/Cy7 (Cat. No. 103116), CD3-PerCP/Cy5.5 (Cat. No. 100328), CD4-Br.Viol421 (Cat. No. 100543), CD8-APC (Cat. No. 100712), NK1.1-PE (Cat. No. 108708), CD11b-Alexa488 (Cat. No. 101217), CD19-PerCP/Cy5.5 (Cat. No. 152406), CD11c-Br.Viol421 (Cat. No. 117329), F4/80-APC (Cat. No. 123116), Ly6G-PE/Cy7 (Cat. No. 127618), MHC2-Alexa 700 (Cat. No. 107622), CD206-PE (Cat. No. 141706). Flow cytometry was performed in the Vanderbilt Medical Center (VUMC) Flow Cytometry Shared Resource. Data were evaluated using FlowJo software v10.6.2 (Tree Star, Ashland, OR, USA). All gates are based on gating from tumor-naïve wildtype mice, and the gating strategy for the major immune cell populations is defined in Figure S3. Flow cytometry data are displayed as the percentage of all CD45+ immune cells unless stated otherwise.

### 4.5. Quantification of Plasma Analytes

At sacrifice, peripheral blood was collected via cardiac puncture into vials coated with ethylenediaminetetraacetic acid (EDTA). Plasma was separated by centrifugation at 1500× *g* and stored at −80 °C until assessed. Immune-related plasma proteins were measured using the R&D Systems Proteome Profiler Mouse XL Cytokine Array Kit (Cat. No. ARY028, R&D Systems, Minneapolis, MN, USA). Normalization and mean pixel intensity were quantified with Quick Spots software v25.6.0.3 (Ideal Eyes Systems Inc., Bountiful, UT, USA).

### 4.6. Statistical Analysis

All flow cytometry analyses were performed in R version 4.3.1 (https://www.r-project.org/, accessed on 16 June 2023). For percent frequency and percent MHC2 and MFI, we conducted various general linear models using the stats package in R (i.e., lm function). To assess the statistical significance of interactions of interest, we used the emmeans package (https://CRAN.R-project.org/package=emmeans, accessed on 18 December 2023) to make post hoc comparisons and *p*-values. All statistical significance was evaluated at (*p* < 0.05).

For datasets collected from major cell-type populations, statistical analyses were performed separately for data collected from each organ. In each model, cell type and either sex or disease status were the independent variables of interest, and we tested for the effects of sex (or disease status) and cell type on cell percent frequency while adjusting for cohort and animal number. We then performed post hoc comparisons for significant interactions of sex and cell type or disease status and cell type on cell percentage.

Further analyses were performed for data collected from subtypes within specific cell types. Here, we performed statistical analyses within each organ and cell type separately. In each model, we tested for the effects of cell subtype and either sex or disease status on cell frequency while adjusting for cohort or animal number. We eliminated the cohort covariate in two tests, as data were collected only for one cohort. Similarly, for one model, we removed the main effect of sex as data were collected for only one sex and thus tested for statistical significance for the main effect of the subtype.

Where applicable, an unpaired *t*-test or two-way ANOVA was performed for disease metrics, immunohistochemistry, and immunoblots experiments using GraphPad Prism 10.1.0 (264) Macintosh Version. In the graphs, error bars represent the standard error of means, and asterisks represent the *p*-values: * *p* ≤ 0.05, ** *p* ≤ 0.01, ***p ≤ 0.001 and *** *p* ≤ 0.0001. All *p*-values less than 0.05 were considered statistically significant.

## Data Availability

Datasets from single-cell RNA sequencing experiments have been deposited in the Gene Expression Omnibus (GEO) publicly available genomics data repository, GSE283609. All other raw data are available upon request from the authors.

## Supplementary Materials

The following supporting information can be downloaded at: https://www.mdpi.com/article/10.3390/ijms252313113/s1.
