# Supplementary material for: Elucidating Sex-Specific Immune Profiles in a Breast Cancer Model
Source: Int J Mol Sci. 2024 Dec 6;25(23):13113. doi: 10.3390/ijms252313113 (PMC11641823; doi:10.3390/ijms252313113)
Supplement: Supplementary file 1 [file ijms-25-13113-s001.zip › ijms-3313629-supplementary.pdf]

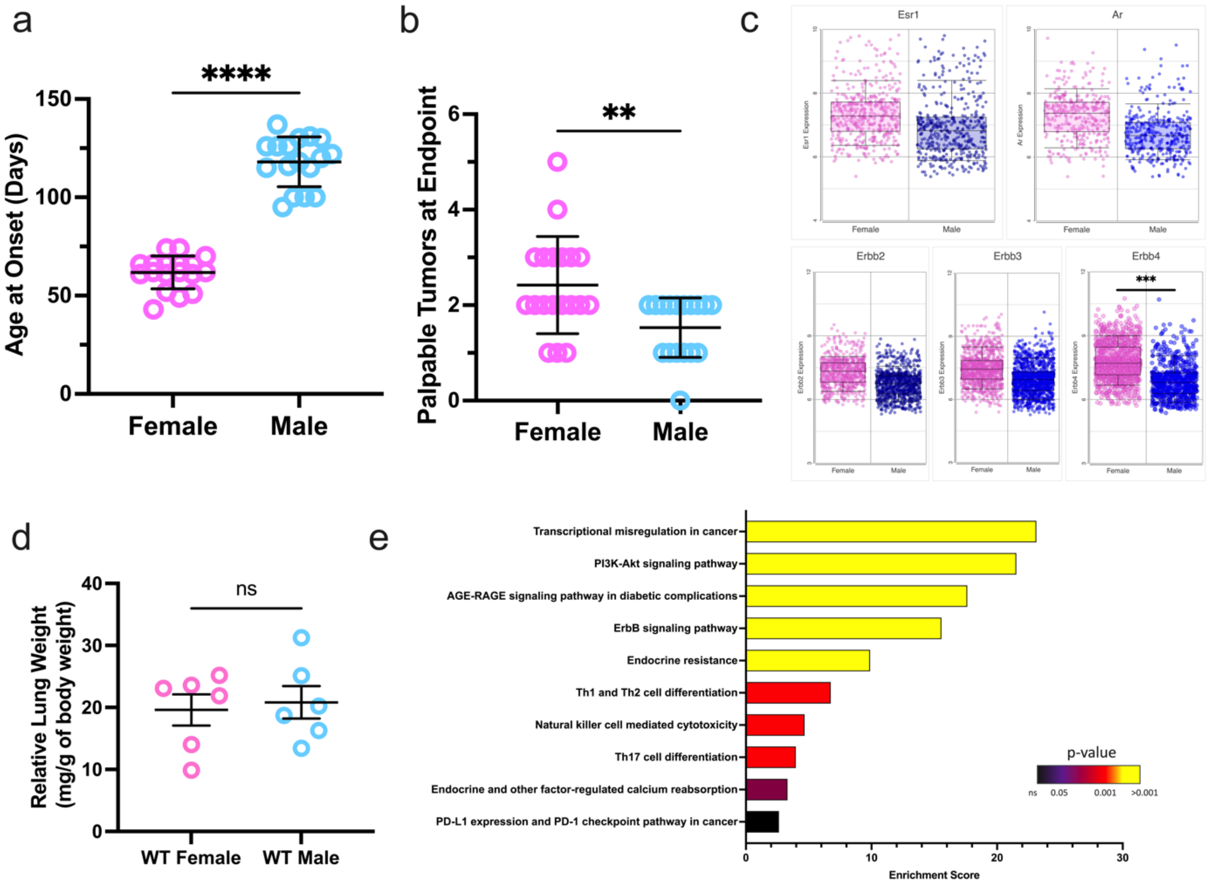

Figure S1: Transgenic mice have differential disease kinetics, but male and female mammary tumors have similar sex steroid levels and epidermal growth factor receptor transcript expression. (a) Palpable tumors were first detected at 61.78 ± 8 days old in transgenic females and 118.1 ± 12 days old in transgenic males; (b) Total number of palpable, primary tumors at endpoint based on sex; (c) Mammary tumors were harvested from each sex. RNA was isolated using the PIPseq T20 3` Single Cell RNA kit. (c) Estrogen receptor alpha (Esr1), androgen receptor (Ar), Errb2, Errb3, and Errb4 transcript levels were compared using the Partek Hurdle Model, similar to an ANOVA test; (d) Lung weights of nontransgenic mice normalized to body weight. All measurements were collected at the study endpoints; (e) Pathway enrichment analysis of biological processes in male versus female tumors.

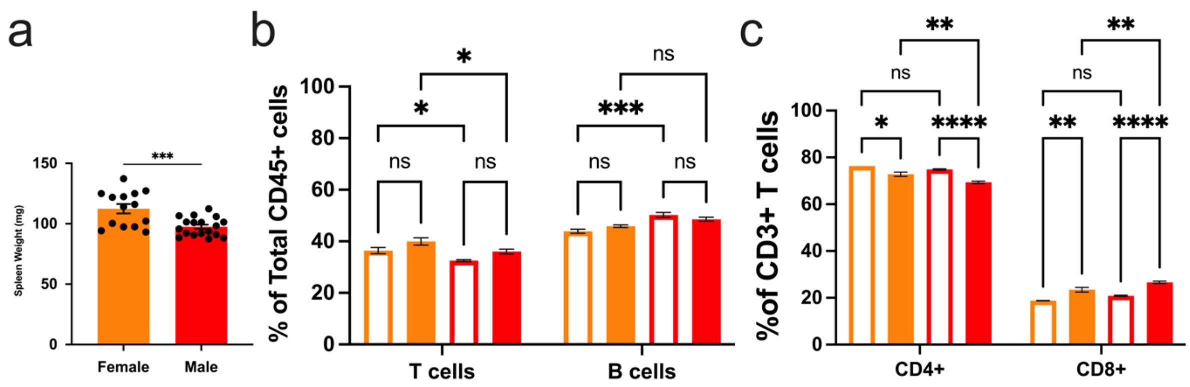

Figure S2: Baseline sex- and age-related differences in tumor naïve mice. (a) Spleen weights of wildtype mice; Splenic immune composition was evaluated using flow cytometry. (b-c) Total T-cells, B-cells and T-cell subsets were assessed in non-transgenic female (orange) and male (red) at 2 months (unfilled) and 5 months (filled) of age.

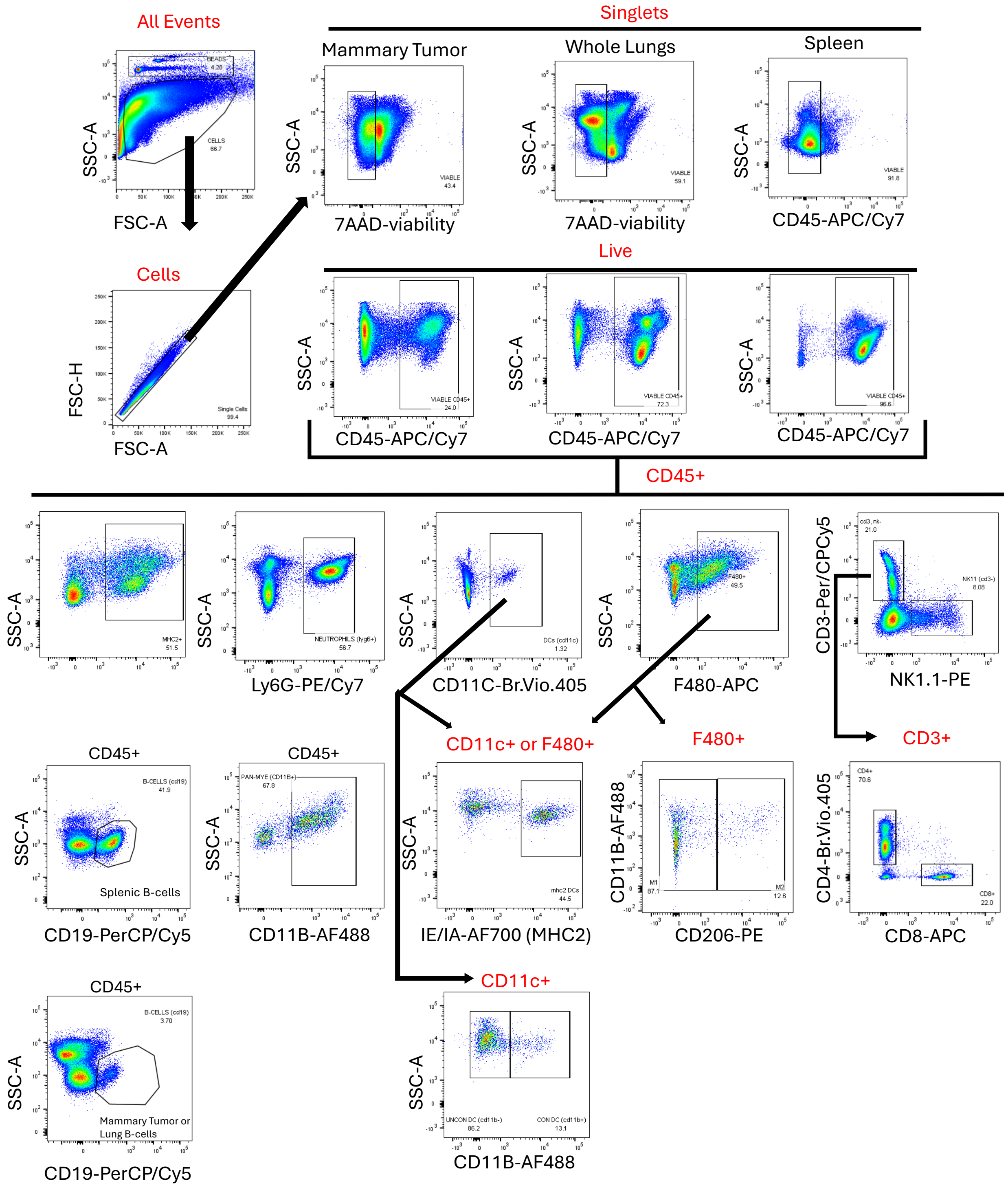

Figure S3. Gating Strategy.

Table S1: **Top 1000 GO terms describing biological processes and molecular functions that are enriched in male versus female tumors.**

Gene set	Description	Enrichment score	P-value	FDR step up	Genes in set	Genes in list
GO:0006139	nucleobase-containing compound metabolic process	734.413	1.11852e-319	9.26048e-317	2404	1924
GO:0046483	heterocycle metabolic process	728.335	4.87911e-317	3.89528e-314	2551	2010
GO:0050794	regulation of cellular process	708.396	0	0	10407	6091
GO:0050789	regulation of biological process	708.396	0	0	10988	6346
GO:0044238	primary metabolic process	708.396	0	0	6668	4705
GO:1901564	organonitrogen compound metabolic process	708.396	0	0	4451	3111
GO:0071704	organic substance metabolic process	708.396	0	0	7329	4983
GO:0006807	nitrogen compound metabolic process	708.396	0	0	6061	4391
GO:0008152	metabolic process	708.396	0	0	7716	5243
GO:0043170	macromolecule metabolic process	708.396	0	0	5554	3948
GO:0009987	cellular process	708.396	0	0	13881	8259
GO:0034641	cellular nitrogen compound metabolic process	708.396	0	0	2888	2291
GO:0044237	cellular metabolic process	708.396	0	0	5708	3962
GO:0071840	cellular component organization or biogenesis	708.396	0	0	5285	3610
GO:0016043	cellular component organization	708.396	0	0	5171	3500
GO:0065007	biological regulation	708.396	0	0	11388	6549
GO:0005515	protein binding	708.396	0	0	9362	5747
GO:0005488	binding	708.396	0	0	13482	7918
GO:0003824	catalytic activity	697.093	1.81E-303	1.30E-300	5494	3616
GO:0006725	cellular aromatic compound metabolic process	680.086	4.39E-296	3.07E-293	2619	2022
GO:1901363	heterocyclic compound binding	666.214	4.64E-290	3.15E-287	5337	3508
GO:0097159	organic cyclic compound binding	660.501	1.41E-287	9.24E-285	5422	3547
GO:0019538	protein metabolic process	644.936	8.09E-281	5.17E-278	3475	2491
GO:1901360	organic cyclic compound metabolic process	635.603	9.14E-277	5.68E-274	2862	2136
GO:0090304	nucleic acid metabolic process	630.392	1.68E-274	1.01E-271	1934	1576
GO:0019222	regulation of metabolic process	627.413	3.30E-273	1.94E-270	6534	4080
GO:0031323	regulation of cellular metabolic process	580.957	4.94E-253	2.76E-250	5905	3720
GO:0080090	regulation of primary metabolic process	569.422	5.05E-248	2.69E-245	5597	3553
GO:0043412	macromolecule modification	563.277	2.35E-245	1.22E-242	2372	1802
GO:0051171	regulation of nitrogen compound metabolic process	561.04	2.20E-244	1.12E-241	5387	3438
GO:0043167	ion binding	553.294	5.10E-241	2.53E-238	5333	3403
GO:0060255	regulation of macromolecule metabolic process	547.807	1.23E-238	5.98E-236	6016	3743
GO:0051641	cellular localization	544.267	4.25E-237	2.02E-234	2293	1743
GO:0048518	positive regulation of biological process	533.726	1.61E-232	7.33E-230	6324	3882
GO:0048522	positive regulation of cellular process	510.675	1.65E-222	7.37E-220	5820	3607
GO:0036211	protein modification process	499.691	9.71E-218	4.25E-215	2197	1657

GO:0048523	negative regulation of cellular process	499.335	1.39E-217	5.96E-215	5031	3196
GO:0016070	RNA metabolic process	487.917	1.26E-212	5.22E-210	1343	1126
GO:0019899	enzyme binding	472.752	4.86E-206	1.97E-203	2344	1724
GO:0048519	negative regulation of biological process	468.153	4.83E-204	1.93E-201	5502	3403
GO:0046907	intracellular transport	449.219	8.07E-196	3.16E-193	1265	1056
GO:0033036	macromolecule localization	439.641	1.17E-191	4.49E-189	1916	1451
GO:0070727	cellular macromolecule localization	432.61	1.32E-188	4.99E-186	1902	1438
GO:0008104	protein localization	432.548	1.40E-188	5.22E-186	1897	1435
GO:0051179	localization	430.355	1.26E-187	4.60E-185	4239	2719
GO:0009889	regulation of biosynthetic process	428.284	9.97E-187	3.59E-184	5039	3127
GO:0003676	nucleic acid binding	427.894	1.47E-186	5.23E-184	3352	2248
GO:0006996	organelle organization	425.777	1.22E-185	4.27E-183	2465	1758
GO:0031326	regulation of cellular biosynthetic process	422.483	3.30E-184	1.13E-181	4996	3099
GO:0010556	regulation of macromolecule biosynthetic process	410.963	3.32E-179	1.12E-176	4841	3008
GO:0009893	positive regulation of metabolic process	409.475	1.47E-178	4.90E-176	3783	2460
GO:0051173	positive regulation of nitrogen compound metabolic process	400.335	1.37E-174	4.50E-172	2989	2027
GO:0003723	RNA binding	394.51	4.64E-172	1.50E-169	1104	924
GO:0043933	protein-containing complex organization	391.456	9.84E-171	3.14E-168	1753	1322
GO:0010468	regulation of gene expression	385.697	3.12E-168	9.68E-166	4714	2916
GO:0010604	positive regulation of macromolecule metabolic process	376.32	3.68E-164	1.11E-161	3437	2245
GO:0006396	RNA processing	372.983	1.04E-162	3.09E-160	785	701
GO:0019219	regulation of nucleobase-containing compound metabolic proc	370.74	9.77E-162	2.87E-159	3771	2415
GO:0022607	cellular component assembly	367.72	2.00E-160	5.81E-158	2199	1562
GO:0051128	regulation of cellular component organization	365.55	1.75E-159	5.02E-157	2577	1771
GO:0033554	cellular response to stress	360.362	3.14E-157	8.88E-155	1486	1142
GO:0051649	establishment of localization in cell	354.591	1.01E-154	2.81E-152	1710	1271
GO:0045184	establishment of protein localization	352.5	8.15E-154	2.25E-151	1253	995
GO:0016740	transferase activity	348.714	3.59E-152	9.79E-150	2230	1564
GO:0044271	cellular nitrogen compound biosynthetic process	348.106	6.60E-152	1.78E-149	1267	1001
GO:0015031	protein transport	341.989	2.99E-149	7.96E-147	1152	926
GO:0031325	positive regulation of cellular metabolic process	338.114	1.44E-147	3.79E-145	3392	2183
GO:0009056	catabolic process	332.128	5.73E-145	1.49E-142	1837	1328
GO:0000166	nucleotide binding	330.357	3.37E-144	8.56E-142	2054	1450
GO:1901265	nucleoside phosphate binding	330.357	3.37E-144	8.56E-142	2054	1450
GO:0046872	metal ion binding	324.92	7.75E-142	1.95E-139	3610	2283
GO:0009892	negative regulation of metabolic process	323.952	2.04E-141	5.06E-139	3005	1964
GO:0031324	negative regulation of cellular metabolic process	321.583	2.18E-140	5.35E-138	2622	1756
GO:0051234	establishment of localization	320.98	3.98E-140	9.68E-138	3624	2286

GO:0051716	cellular response to stimulus	317.489	1.31E-138	3.11E-136	2744	1818
GO:0043169	cation binding	316.897	2.36E-138	5.56E-136	3708	2325
GO:0009058	biosynthetic process	316.07	5.40E-138	1.26E-135	2567	1721
GO:0051252	regulation of RNA metabolic process	315.571	8.90E-138	2.05E-135	3489	2210
GO:1901576	organic substance biosynthetic process	311.569	4.87E-136	1.11E-133	2514	1688
GO:0071705	nitrogen compound transport	304.735	4.52E-133	1.02E-130	1570	1153
GO:0051246	regulation of protein metabolic process	303.262	1.97E-132	4.41E-130	2396	1616
GO:0044249	cellular biosynthetic process	298.466	2.39E-130	5.28E-128	2373	1599
GO:0009057	macromolecule catabolic process	298.053	3.61E-130	7.91E-128	955	777
GO:0006810	transport	297.413	6.84E-130	1.48E-127	3456	2173
GO:0010605	negative regulation of macromolecule metabolic process	294.226	1.66E-128	3.56E-126	2770	1809
GO:0017076	purine nucleotide binding	292.733	7.37E-128	1.57E-125	1898	1331
GO:0140096	catalytic activity, acting on a protein	290.351	7.98E-127	1.68E-124	2266	1533
GO:0044877	protein-containing complex binding	288.973	3.17E-126	6.62E-124	2121	1452
GO:0036094	small molecule binding	284.67	2.34E-124	4.84E-122	2469	1638
GO:0016071	mRNA metabolic process	284.599	2.51E-124	5.15E-122	564	511
GO:0006793	phosphorus metabolic process	282.438	2.18E-123	4.39E-121	1729	1227
GO:0035639	purine ribonucleoside triphosphate binding	279.896	2.77E-122	5.53E-120	1708	1213
GO:0043168	anion binding	279.631	3.61E-122	7.15E-120	2345	1566
GO:1901575	organic substance catabolic process	279.482	4.19E-122	8.23E-120	1593	1147
GO:0032553	ribonucleotide binding	273.97	1.04E-119	2.00E-117	1807	1264
GO:0006796	phosphate-containing compound metabolic process	273.8	1.23E-119	2.35E-117	1708	1208
GO:0032555	purine ribonucleotide binding	271.753	9.53E-119	1.81E-116	1789	1252
GO:0043687	post-translational protein modification	264.732	1.07E-115	2.00E-113	819	672
GO:0045935	positive regulation of nucleobase-containing compound metab	261.951	1.72E-114	3.21E-112	1919	1316
GO:0051172	negative regulation of nitrogen compound metabolic process	258.843	3.85E-113	7.12E-111	2353	1550
GO:2001141	regulation of RNA biosynthetic process	256.819	2.92E-112	5.35E-110	3205	1997
GO:0006355	regulation of DNA-templated transcription	255.243	1.41E-111	2.56E-109	3195	1990
GO:0048583	regulation of response to stimulus	249.992	2.69E-109	4.85E-107	4004	2396
GO:0006974	DNA damage response	247.93	2.12E-108	3.78E-106	718	598
GO:0009890	negative regulation of biosynthetic process	245.693	1.98E-107	3.51E-105	2179	1443
GO:0033043	regulation of organelle organization	244.301	7.97E-107	1.39E-104	1190	886
GO:0031327	negative regulation of cellular biosynthetic process	243.116	2.61E-106	4.52E-104	2156	1428
GO:0010558	negative regulation of macromolecule biosynthetic process	240.05	5.59E-105	9.62E-103	2097	1393
GO:0030554	adenyl nucleotide binding	239.696	7.97E-105	1.36E-102	1564	1098
GO:0097367	carbohydrate derivative binding	237.999	4.35E-104	7.37E-102	2171	1431
GO:0006886	intracellular protein transport	237.875	4.92E-104	8.27E-102	663	557
GO:0009894	regulation of catabolic process	237.005	1.18E-103	1.96E-101	958	742

GO:0009966	regulation of signal transduction	232.585	9.77E-102	1.62E-99	2997	1861
GO:0023051	regulation of signaling	232.062	1.65E-101	2.71E-99	3470	2103
GO:0071702	organic substance transport	231.712	2.34E-101	3.82E-99	2129	1402
GO:1901566	organonitrogen compound biosynthetic process	227.616	1.41E-99	2.28E-97	927	717
GO:0042802	identical protein binding	226.849	3.02E-99	4.86E-97	2372	1527
GO:0010646	regulation of cell communication	225.775	8.85E-99	1.41E-96	3470	2095
GO:0009891	positive regulation of biosynthetic process	225.35	1.35E-98	2.15E-96	2673	1684
GO:0005524	ATP binding	223.645	7.45E-98	1.17E-95	1385	983
GO:0031328	positive regulation of cellular biosynthetic process	222.381	2.64E-97	4.12E-95	2647	1667
GO:0032559	adenyl ribonucleotide binding	219.764	3.61E-96	5.57E-94	1456	1020
GO:0010557	positive regulation of macromolecule biosynthetic process	219.233	6.14E-96	9.40E-94	2547	1611
GO:0051254	positive regulation of RNA metabolic process	216.102	1.41E-94	2.11E-92	1713	1160
GO:0051726	regulation of cell cycle	212.356	5.95E-93	8.87E-91	1060	786
GO:0006397	mRNA processing	211.701	1.15E-92	1.70E-90	412	375
GO:0034660	ncRNA metabolic process	210.988	2.34E-92	3.44E-90	477	420
GO:0071824	protein-DNA complex organization	210.047	5.99E-92	8.76E-90	707	570
GO:0006950	response to stress	206.685	1.73E-90	2.51E-88	3004	1833
GO:0065003	protein-containing complex assembly	206.096	3.12E-90	4.49E-88	1097	803
GO:0043632	modification-dependent macromolecule catabolic process	203.419	4.53E-89	6.49E-87	557	470
GO:0006412	translation	201.493	3.11E-88	4.43E-86	300	289
GO:0051603	proteolysis involved in protein catabolic process	200.199	1.13E-87	1.60E-85	637	520
GO:0065009	regulation of molecular function	200.038	1.33E-87	1.87E-85	2513	1571
GO:0018130	heterocycle biosynthetic process	197.275	2.11E-86	2.95E-84	830	638
GO:0016192	vesicle-mediated transport	197.111	2.49E-86	3.45E-84	1109	803
GO:0019941	modification-dependent protein catabolic process	196.674	3.85E-86	5.31E-84	547	460
GO:0016787	hydrolase activity	194.771	2.58E-85	3.54E-83	2328	1468
GO:0044087	regulation of cellular component biogenesis	194.211	4.52E-85	6.12E-83	1015	746
GO:0070647	protein modification by small protein conjugation or removal	189.785	3.78E-83	5.06E-81	656	526
GO:0006511	ubiquitin-dependent protein catabolic process	188.633	1.20E-82	1.59E-80	535	448
GO:0051247	positive regulation of protein metabolic process	187.204	4.99E-82	6.57E-80	1388	953
GO:1902680	positive regulation of RNA biosynthetic process	186.529	9.80E-82	1.27E-79	1601	1070
GO:0045893	positive regulation of DNA-templated transcription	185.154	3.88E-81	5.01E-79	1598	1067
GO:0034654	nucleobase-containing compound biosynthetic process	184.89	5.05E-81	6.45E-79	758	586
GO:0043043	peptide biosynthetic process	184.137	1.07E-80	1.36E-78	322	300
GO:0008380	RNA splicing	182.824	3.99E-80	5.04E-78	324	301
GO:0043067	regulation of programmed cell death	182.601	4.98E-80	6.25E-78	1643	1089
GO:0006259	DNA metabolic process	182.418	5.98E-80	7.47E-78	697	547
GO:0034470	ncRNA processing	181.585	1.38E-79	1.71E-77	388	346

GO:0043603	amide metabolic process	181.407	1.64E-79	2.03E-77	677	534
GO:0033365	protein localization to organelle	181.025	2.41E-79	2.96E-77	679	535
GO:0006518	peptide metabolic process	179.761	8.53E-79	1.04E-76	408	359
GO:0019438	aromatic compound biosynthetic process	179.595	1.01E-78	1.22E-76	842	633
GO:0006325	chromatin organization	178.573	2.80E-78	3.36E-76	638	508
GO:0042981	regulation of apoptotic process	177.338	9.62E-78	1.15E-75	1594	1057
GO:0003712	transcription coregulator activity	176.513	2.20E-77	2.61E-75	488	411
GO:0043604	amide biosynthetic process	173.437	4.76E-76	5.63E-74	427	369
GO:0140640	catalytic activity, acting on a nucleic acid	171.752	2.56E-75	3.02E-73	576	465
GO:0009059	macromolecule biosynthetic process	170.78	6.78E-75	7.93E-73	1487	992
GO:0031399	regulation of protein modification process	169.992	1.49E-74	1.73E-72	1401	944
GO:0060341	regulation of cellular localization	168.611	5.93E-74	6.87E-72	1087	767
GO:0080135	regulation of cellular response to stress	168.462	6.89E-74	7.93E-72	666	519
GO:0044248	cellular catabolic process	167.448	1.90E-73	2.18E-71	984	707
GO:0045934	negative regulation of nucleobase-containing compound meta	166.706	3.99E-73	4.55E-71	1511	1001
GO:1901362	organic cyclic compound biosynthetic process	165.985	8.20E-73	9.30E-71	967	696
GO:0032502	developmental process	162.772	2.04E-71	2.30E-69	5343	2918
GO:0006357	regulation of transcription by RNA polymerase II	162.633	2.34E-71	2.63E-69	2448	1491
GO:0008134	transcription factor binding	162.47	2.75E-71	3.08E-69	712	543
GO:1902531	regulation of intracellular signal transduction	160.789	1.48E-70	1.65E-68	1721	1108
GO:0050793	regulation of developmental process	160.447	2.08E-70	2.30E-68	2643	1588
GO:0035556	intracellular signal transduction	160.343	2.31E-70	2.54E-68	1455	964
GO:0019900	kinase binding	158.946	9.35E-70	1.02E-67	887	644
GO:1901565	organonitrogen compound catabolic process	158.906	9.73E-70	1.06E-67	834	613
GO:0006915	apoptotic process	158.052	2.28E-69	2.47E-67	830	610
GO:0006281	DNA repair	156.771	8.23E-69	8.80E-67	464	385
GO:0032446	protein modification by small protein conjugation	155.963	1.85E-68	1.95E-66	540	433
GO:0051130	positive regulation of cellular component organization	155.703	2.39E-68	2.52E-66	1263	854
GO:0030163	protein catabolic process	155.659	2.50E-68	2.62E-66	526	424
GO:0010564	regulation of cell cycle process	153.669	1.83E-67	1.90E-65	729	547
GO:0019904	protein domain specific binding	153.56	2.04E-67	2.11E-65	812	596
GO:0016772	transferase activity, transferring phosphorus-containing groups	153.148	3.08E-67	3.17E-65	895	644
GO:0050790	regulation of catalytic activity	153.036	3.45E-67	3.53E-65	1743	1111
GO:0022402	cell cycle process	152.579	5.44E-67	5.56E-65	863	625
GO:0032880	regulation of protein localization	150.358	5.01E-66	5.10E-64	981	691
GO:0012501	programmed cell death	149.66	1.01E-65	1.02E-63	893	640
GO:0008219	cell death	148.958	2.03E-65	2.05E-63	894	640
GO:0010608	post-transcriptional regulation of gene expression	147.808	6.42E-65	6.44E-63	467	382

GO:0044093	positive regulation of molecular function	147.7	7.16E-65	7.14E-63	1472	960
GO:1903047	mitotic cell cycle process	147.234	1.14E-64	1.13E-62	499	402
GO:0051253	negative regulation of RNA metabolic process	145.502	6.44E-64	6.37E-62	1395	916
GO:0019637	organophosphate metabolic process	144.423	1.90E-63	1.87E-61	826	597
GO:0019901	protein kinase binding	143.94	3.07E-63	3.01E-61	794	578
GO:0010629	negative regulation of gene expression	143.625	4.21E-63	4.11E-61	1002	697
GO:0009968	negative regulation of signal transduction	142.404	1.43E-62	1.39E-60	1309	866
GO:0003677	DNA binding	140.482	9.76E-62	9.40E-60	2299	1385
GO:0006364	rRNA processing	138.717	5.70E-61	5.45E-59	204	197
GO:0010498	proteasomal protein catabolic process	138.582	6.53E-61	6.21E-59	379	320
GO:0048193	Golgi vesicle transport	137.354	2.23E-60	2.10E-58	246	228
GO:0016072	rRNA metabolic process	136.594	4.77E-60	4.44E-58	230	216
GO:0003682	chromatin binding	136.471	5.39E-60	5.00E-58	661	494
GO:0048585	negative regulation of response to stimulus	136.205	7.03E-60	6.50E-58	1737	1088
GO:0018193	peptidyl-amino acid modification	135.799	1.05E-59	9.70E-58	635	478
GO:0034248	regulation of amide metabolic process	133.82	7.64E-59	6.97E-57	434	353
GO:0090407	organophosphate biosynthetic process	133.228	1.38E-58	1.25E-56	463	371
GO:0016567	protein ubiquitination	132.623	2.53E-58	2.29E-56	494	390
GO:0031329	regulation of cellular catabolic process	131.905	5.18E-58	4.67E-56	583	444
GO:0042127	regulation of cell population proliferation	131.454	8.13E-58	7.30E-56	2357	1402
GO:0023057	negative regulation of signaling	130.466	2.18E-57	1.95E-55	1431	919
GO:0051301	cell division	130.257	2.69E-57	2.39E-55	442	356
GO:0007005	mitochondrion organization	130.206	2.83E-57	2.50E-55	343	292
GO:0044281	small molecule metabolic process	129.237	7.47E-57	6.55E-55	1569	991
GO:0044085	cellular component biogenesis	129.135	8.27E-57	7.22E-55	183	178
GO:0009896	positive regulation of catabolic process	128.227	2.05E-56	1.78E-54	505	394
GO:0030234	enzyme regulator activity	126.862	8.03E-56	6.96E-54	1236	810
GO:0006338	chromatin remodeling	126.857	8.07E-56	6.97E-54	458	364
GO:0010648	negative regulation of cell communication	126.429	1.24E-55	1.06E-53	1434	916
GO:0016746	acyltransferase activity	126.101	1.72E-55	1.47E-53	705	512
GO:0006417	regulation of translation	125.661	2.67E-55	2.28E-53	371	308
GO:0022613	ribonucleoprotein complex biogenesis	125.631	2.75E-55	2.34E-53	169	166
GO:0051129	negative regulation of cellular component organization	125.403	3.45E-55	2.92E-53	756	541
GO:0060090	molecular adaptor activity	125.26	3.98E-55	3.36E-53	616	459
GO:0051052	regulation of DNA metabolic process	123.622	2.05E-54	1.72E-52	528	405
GO:0006508	proteolysis	123.173	3.21E-54	2.69E-52	1223	799
GO:0140297	DNA-binding transcription factor binding	122.385	7.06E-54	5.89E-52	536	409
GO:1902679	negative regulation of RNA biosynthetic process	122.183	8.65E-54	7.18E-52	1288	833

GO:0080134	regulation of response to stress	122.171	8.75E-54	7.24E-52	1498	945
GO:0045892	negative regulation of DNA-templated transcription	121.556	1.62E-53	1.33E-51	1280	828
GO:0043161	proteasome-mediated ubiquitin-dependent protein catabolic p	121.552	1.62E-53	1.33E-51	336	283
GO:0051020	GTPase binding	121.498	1.71E-53	1.40E-51	333	281
GO:0051248	negative regulation of protein metabolic process	120.602	4.20E-53	3.43E-51	994	671
GO:0140098	catalytic activity, acting on RNA	117.996	5.69E-52	4.60E-50	362	298
GO:0070925	organelle assembly	117.629	8.21E-52	6.62E-50	636	465
GO:1901987	regulation of cell cycle phase transition	116.689	2.10E-51	1.69E-49	428	339
GO:0003735	structural constituent of ribosome	115.982	4.26E-51	3.41E-49	162	158
GO:1901873	regulation of post-translational protein modification	114.497	1.88E-50	1.50E-48	293	251
GO:0045944	positive regulation of transcription by RNA polymerase II	114.272	2.36E-50	1.86E-48	1258	808
GO:0016310	phosphorylation	114.14	2.69E-50	2.12E-48	849	584
GO:0007346	regulation of mitotic cell cycle	113.4	5.64E-50	4.42E-48	500	381
GO:0010638	positive regulation of organelle organization	113.046	8.03E-50	6.27E-48	527	397
GO:0042176	regulation of protein catabolic process	111.398	4.17E-49	3.25E-47	360	293
GO:0043085	positive regulation of catalytic activity	111.25	4.84E-49	3.75E-47	1044	689
GO:1901135	carbohydrate derivative metabolic process	111.015	6.12E-49	4.74E-47	776	540
GO:0061024	membrane organization	110.977	6.36E-49	4.90E-47	575	424
GO:0007049	cell cycle	110.259	1.30E-48	1.00E-46	843	577
GO:0051174	regulation of phosphorus metabolic process	110.067	1.58E-48	1.21E-46	1314	833
GO:0008092	cytoskeletal protein binding	109.984	1.72E-48	1.31E-46	1008	668
GO:0050896	response to stimulus	109.702	2.27E-48	1.73E-46	5439	2858
GO:0019220	regulation of phosphate metabolic process	109.673	2.34E-48	1.78E-46	1313	832
GO:0000375	RNA splicing, via transesterification reactions	109.181	3.83E-48	2.89E-46	171	163
GO:0051668	localization within membrane	109.12	4.07E-48	3.07E-46	552	409
GO:0016817	hydrolase activity, acting on acid anhydrides	108.991	4.63E-48	3.47E-46	641	461
GO:0044389	ubiquitin-like protein ligase binding	108.719	6.08E-48	4.53E-46	353	287
GO:0016818	hydrolase activity, acting on acid anhydrides, in phosphorus-co	108.479	7.73E-48	5.74E-46	640	460
GO:0000377	RNA splicing, via transesterification reactions with bulged aden	108.397	8.39E-48	6.19E-46	170	162
GO:0000398	mRNA splicing, via spliceosome	108.397	8.39E-48	6.19E-46	170	162
GO:0008283	cell population proliferation	107.967	1.29E-47	9.48E-46	1872	1121
GO:0031267	small GTPase binding	107.848	1.45E-47	1.06E-45	295	249
GO:0016301	kinase activity	107.448	2.17E-47	1.58E-45	734	513
GO:0072594	establishment of protein localization to organelle	106.96	3.53E-47	2.57E-45	328	270
GO:0060589	nucleoside-triphosphatase regulator activity	106.7	4.58E-47	3.31E-45	427	332
GO:0030695	GTPase regulator activity	106.7	4.58E-47	3.31E-45	427	332
GO:0003713	transcription coactivator activity	106.39	6.25E-47	4.50E-45	263	227
GO:0016462	pyrophosphatase activity	105.417	1.65E-46	1.19E-44	634	454

GO:0043069	negative regulation of programmed cell death	105.394	1.69E-46	1.21E-44	1004	661
GO:0048856	anatomical structure development	104.913	2.74E-46	1.95E-44	3729	2035
GO:0019787	ubiquitin-like protein transferase activity	103.99	6.88E-46	4.90E-44	448	343
GO:0071826	protein-RNA complex organization	103.74	8.84E-46	6.27E-44	172	162
GO:1902532	negative regulation of intracellular signal transduction	103.708	9.13E-46	6.46E-44	571	416
GO:0061919	process utilizing autophagic mechanism	103.613	1.00E-45	7.05E-44	265	227
GO:0006914	autophagy	103.613	1.00E-45	7.05E-44	265	227
GO:0031647	regulation of protein stability	103.263	1.42E-45	9.94E-44	311	257
GO:0055086	nucleobase-containing small molecule metabolic process	102.477	3.12E-45	2.16E-43	493	369
GO:0031625	ubiquitin protein ligase binding	101.954	5.27E-45	3.64E-43	334	271
GO:0051338	regulation of transferase activity	101.736	6.56E-45	4.51E-43	777	532
GO:0016755	aminoacyltransferase activity	101.606	7.46E-45	5.12E-43	464	351
GO:0032879	regulation of localization	101.289	1.02E-44	7.00E-43	2315	1335
GO:0043066	negative regulation of apoptotic process	101.023	1.34E-44	9.12E-43	974	640
GO:0051493	regulation of cytoskeleton organization	100.629	1.98E-44	1.35E-42	524	386
GO:0007010	cytoskeleton organization	100.162	3.16E-44	2.14E-42	950	626
GO:0043021	ribonucleoprotein complex binding	99.5123	6.06E-44	4.09E-42	185	170
GO:1901990	regulation of mitotic cell cycle phase transition	98.3697	1.90E-43	1.28E-41	331	267
GO:1901137	carbohydrate derivative biosynthetic process	97.8852	3.08E-43	2.07E-41	399	309
GO:0045595	regulation of cell differentiation	97.6784	3.79E-43	2.54E-41	1688	1012
GO:0022618	protein-RNA complex assembly	97.571	4.22E-43	2.82E-41	164	154
GO:0016197	endosomal transport	97.5602	4.27E-43	2.84E-41	216	191
GO:0016773	phosphotransferase activity, alcohol group as acceptor	96.8496	8.68E-43	5.76E-41	682	474
GO:0070887	cellular response to chemical stimulus	96.392	1.37E-42	9.08E-41	1639	985
GO:0008047	enzyme activator activity	96.2096	1.65E-42	1.09E-40	547	396
GO:0048584	positive regulation of response to stimulus	95.5071	3.33E-42	2.19E-40	2300	1318
GO:0043254	regulation of protein-containing complex assembly	95.2192	4.43E-42	2.91E-40	430	326
GO:0009967	positive regulation of signal transduction	94.5161	8.96E-42	5.85E-40	1615	970
GO:0006399	tRNA metabolic process	93.7298	1.97E-41	1.28E-39	159	149
GO:0042221	response to chemical	93.4939	2.49E-41	1.62E-39	2642	1484
GO:0004842	ubiquitin-protein transferase activity	92.7049	5.48E-41	3.55E-39	423	320
GO:0031344	regulation of cell projection organization	92.7006	5.50E-41	3.56E-39	782	526
GO:0008270	zinc ion binding	92.4566	7.02E-41	4.50E-39	707	484
GO:0042325	regulation of phosphorylation	91.9246	1.20E-40	7.64E-39	1163	731
GO:0023056	positive regulation of signaling	91.5641	1.71E-40	1.09E-38	1845	1083
GO:0051239	regulation of multicellular organismal process	91.5265	1.78E-40	1.13E-38	3225	1765
GO:0031401	positive regulation of protein modification process	91.523	1.79E-40	1.13E-38	923	602
GO:1903320	regulation of protein modification by small protein conjugation	91.4759	1.87E-40	1.18E-38	247	209

GO:0061629	RNA polymerase II-specific DNA-binding transcription factor bir	89.8691	9.34E-40	5.88E-38	380	292
GO:0010506	regulation of autophagy	89.7166	1.09E-39	6.83E-38	298	241
GO:0010647	positive regulation of cell communication	89.6333	1.18E-39	7.40E-38	1843	1079
GO:0032774	RNA biosynthetic process	89.4234	1.46E-39	9.11E-38	464	342
GO:1903829	positive regulation of protein localization	88.4056	4.04E-39	2.51E-37	527	378
GO:0120035	regulation of plasma membrane bounded cell projection organ	88.369	4.19E-39	2.60E-37	770	515
GO:0003729	mRNA binding	88.166	5.13E-39	3.18E-37	333	262
GO:1903050	regulation of proteolysis involved in protein catabolic process	87.3229	1.19E-38	7.34E-37	226	193
GO:0032386	regulation of intracellular transport	87.2873	1.23E-38	7.58E-37	342	267
GO:0016788	hydrolase activity, acting on ester bonds	86.7272	2.16E-38	1.32E-36	735	494
GO:0017111	ribonucleoside triphosphate phosphatase activity	86.559	2.56E-38	1.56E-36	575	404
GO:0006913	nucleocytoplasmic transport	86.4651	2.81E-38	1.71E-36	251	209
GO:0051169	nuclear transport	86.4651	2.81E-38	1.71E-36	251	209
GO:0051640	organelle localization	86.1517	3.84E-38	2.32E-36	482	350
GO:0031400	negative regulation of protein modification process	85.9346	4.78E-38	2.88E-36	455	334
GO:0022411	cellular component disassembly	85.7216	5.91E-38	3.55E-36	264	217
GO:0046914	transition metal ion binding	85.6405	6.41E-38	3.84E-36	993	633
GO:0033044	regulation of chromosome organization	84.9867	1.23E-37	7.35E-36	243	203
GO:0044089	positive regulation of cellular component biogenesis	84.9362	1.30E-37	7.71E-36	546	386
GO:0030030	cell projection organization	84.4664	2.07E-37	1.23E-35	1158	719
GO:0006753	nucleoside phosphate metabolic process	84.3722	2.28E-37	1.35E-35	435	321
GO:0034655	nucleobase-containing compound catabolic process	84.1956	2.72E-37	1.60E-35	256	211
GO:0072657	protein localization to membrane	83.7722	4.15E-37	2.44E-35	475	344
GO:0065008	regulation of biological quality	83.2166	7.24E-37	4.25E-35	2732	1509
GO:0051054	positive regulation of DNA metabolic process	82.9673	9.28E-37	5.43E-35	319	250
GO:0070201	regulation of establishment of protein localization	82.8869	1.01E-36	5.87E-35	602	416
GO:0009117	nucleotide metabolic process	82.7108	1.20E-36	6.99E-35	427	315
GO:0000209	protein polyubiquitination	82.0739	2.27E-36	1.32E-34	227	191
GO:0010639	negative regulation of organelle organization	81.3567	4.65E-36	2.69E-34	365	277
GO:1902903	regulation of supramolecular fiber organization	81.0849	6.10E-36	3.52E-34	392	293
GO:1902115	regulation of organelle assembly	80.8215	7.94E-36	4.55E-34	228	191
GO:0006468	protein phosphorylation	80.6955	9.00E-36	5.15E-34	699	468
GO:0140993	histone modifying activity	80.6385	9.53E-36	5.44E-34	195	169
GO:2001233	regulation of apoptotic signaling pathway	79.9852	1.83E-35	1.04E-33	434	317
GO:0030674	protein-macromolecule adaptor activity	79.7671	2.28E-35	1.29E-33	436	318
GO:0061136	regulation of proteasomal protein catabolic process	79.3133	3.59E-35	2.02E-33	190	165
GO:0030334	regulation of cell migration	79.2335	3.88E-35	2.18E-33	1000	629
GO:2000145	regulation of cell motility	78.7474	6.32E-35	3.54E-33	1056	658

GO:0007165	signal transduction	77.157	3.10E-34	1.72E-32	3844	2031
GO:0001932	regulation of protein phosphorylation	76.9824	3.69E-34	2.05E-32	1066	661
GO:0032543	mitochondrial translation	76.8612	4.16E-34	2.30E-32	93	93
GO:0006605	protein targeting	76.4829	6.08E-34	3.35E-32	198	169
GO:0004672	protein kinase activity	76.426	6.44E-34	3.53E-32	579	397
GO:0042254	ribosome biogenesis	76.2828	7.43E-34	4.07E-32	108	105
GO:0040008	regulation of growth	75.9115	1.08E-33	5.87E-32	668	446
GO:0018205	peptidyl-lysine modification	75.4732	1.67E-33	9.08E-32	172	151
GO:0007034	vacuolar transport	75.3878	1.82E-33	9.86E-32	159	142
GO:0071310	cellular response to organic substance	75.0095	2.65E-33	1.43E-31	1269	764
GO:0040012	regulation of locomotion	74.8336	3.16E-33	1.70E-31	1100	676
GO:0000122	negative regulation of transcription by RNA polymerase II	74.45	4.64E-33	2.49E-31	1000	623
GO:0016050	vesicle organization	74.2313	5.78E-33	3.10E-31	278	219
GO:0061659	ubiquitin-like protein ligase activity	74.1614	6.20E-33	3.31E-31	357	267
GO:0010033	response to organic substance	73.8946	8.09E-33	4.32E-31	2250	1255
GO:0046700	heterocycle catabolic process	73.8121	8.79E-33	4.67E-31	293	228
GO:0044270	cellular nitrogen compound catabolic process	73.8121	8.79E-33	4.67E-31	293	228
GO:0051223	regulation of protein transport	73.6683	1.01E-32	5.36E-31	573	391
GO:0031396	regulation of protein ubiquitination	73.4643	1.24E-32	6.56E-31	211	176
GO:0051098	regulation of binding	73.3722	1.36E-32	7.18E-31	402	293
GO:0043549	regulation of kinase activity	73.3064	1.46E-32	7.64E-31	662	440
GO:0097190	apoptotic signaling pathway	73.1545	1.70E-32	8.88E-31	315	241
GO:0045862	positive regulation of proteolysis	72.6013	2.95E-32	1.54E-30	364	270
GO:0072521	purine-containing compound metabolic process	72.5289	3.17E-32	1.65E-30	410	297
GO:0009628	response to abiotic stimulus	72.4604	3.40E-32	1.76E-30	965	602
GO:1903311	regulation of mRNA metabolic process	72.2284	4.28E-32	2.22E-30	272	214
GO:0043065	positive regulation of apoptotic process	71.7812	6.70E-32	3.46E-30	634	423
GO:0043484	regulation of RNA splicing	71.7422	6.96E-32	3.59E-30	182	156
GO:0050821	protein stabilization	71.2079	1.19E-31	6.12E-30	196	165
GO:0043068	positive regulation of programmed cell death	71.1577	1.25E-31	6.42E-30	657	435
GO:0004674	protein serine/threonine kinase activity	70.8609	1.68E-31	8.62E-30	440	313
GO:2000058	regulation of ubiquitin-dependent protein catabolic process	70.4523	2.53E-31	1.29E-29	171	148
GO:0046983	protein dimerization activity	70.4487	2.54E-31	1.30E-29	1073	656
GO:0006091	generation of precursor metabolites and energy	70.4229	2.60E-31	1.33E-29	300	230
GO:0045732	positive regulation of protein catabolic process	70.1213	3.52E-31	1.79E-29	203	169
GO:0034976	response to endoplasmic reticulum stress	70.0388	3.82E-31	1.94E-29	217	178
GO:0019439	aromatic compound catabolic process	69.7665	5.02E-31	2.53E-29	309	235
GO:0044092	negative regulation of molecular function	69.1419	9.38E-31	4.70E-29	996	614

GO:0007033	vacuole organization	69.1288	9.50E-31	4.75E-29	147	131
GO:0032259	methylation	68.9948	1.09E-30	5.42E-29	240	192
GO:0006979	response to oxidative stress	68.9306	1.16E-30	5.77E-29	335	250
GO:0031346	positive regulation of cell projection organization	68.8504	1.26E-30	6.23E-29	457	321
GO:0006401	RNA catabolic process	68.8021	1.32E-30	6.53E-29	153	135
GO:0010563	negative regulation of phosphorus metabolic process	68.69	1.47E-30	7.24E-29	412	295
GO:0045936	negative regulation of phosphate metabolic process	68.69	1.47E-30	7.24E-29	412	295
GO:0007030	Golgi organization	68.4116	1.95E-30	9.54E-29	122	113
GO:0009895	negative regulation of catabolic process	68.1722	2.47E-30	1.21E-28	341	253
GO:0008284	positive regulation of cell population proliferation	67.9099	3.21E-30	1.57E-28	1415	828
GO:0006163	purine nucleotide metabolic process	67.9075	3.22E-30	1.57E-28	377	274
GO:0009141	nucleoside triphosphate metabolic process	67.8951	3.26E-30	1.59E-28	142	127
GO:0140110	transcription regulator activity	67.3688	5.52E-30	2.67E-28	1763	1002
GO:0001558	regulation of cell growth	67.2326	6.33E-30	3.05E-28	442	311
GO:0016570	histone modification	67.2277	6.36E-30	3.06E-28	131	119
GO:0045786	negative regulation of cell cycle	67.2104	6.47E-30	3.10E-28	349	257
GO:0140097	catalytic activity, acting on DNA	66.5603	1.24E-29	5.92E-28	236	188
GO:0010243	response to organonitrogen compound	66.5097	1.30E-29	6.21E-28	729	469
GO:0031331	positive regulation of cellular catabolic process	66.4097	1.44E-29	6.85E-28	308	232
GO:0006282	regulation of DNA repair	66.2458	1.70E-29	8.04E-28	211	172
GO:0008033	tRNA processing	66.1532	1.86E-29	8.80E-28	119	110
GO:0022603	regulation of anatomical structure morphogenesis	66.0356	2.09E-29	9.88E-28	939	580
GO:0008285	negative regulation of cell population proliferation	66.0226	2.12E-29	9.99E-28	920	570
GO:1901875	positive regulation of post-translational protein modification	65.7087	2.90E-29	1.36E-27	176	149
GO:0061630	ubiquitin protein ligase activity	65.5589	3.37E-29	1.58E-27	341	251
GO:1901698	response to nitrogen compound	65.4776	3.66E-29	1.71E-27	806	509
GO:0042803	protein homodimerization activity	65.1975	4.84E-29	2.25E-27	786	498
GO:0009165	nucleotide biosynthetic process	65.1444	5.11E-29	2.37E-27	212	172
GO:2000026	regulation of multicellular organismal development	65.0515	5.60E-29	2.59E-27	1579	906
GO:0032182	ubiquitin-like protein binding	64.9779	6.03E-29	2.79E-27	121	111
GO:0120036	plasma membrane bounded cell projection organization	64.7835	7.33E-29	3.37E-27	925	571
GO:1901293	nucleoside phosphate biosynthetic process	64.6829	8.10E-29	3.72E-27	214	173
GO:0006351	DNA-templated transcription	64.4229	1.05E-28	4.81E-27	394	281
GO:0010948	negative regulation of cell cycle process	63.5861	2.43E-28	1.11E-26	265	204
GO:0009719	response to endogenous stimulus	63.3794	2.98E-28	1.35E-26	963	589
GO:0033108	mitochondrial respiratory chain complex assembly	63.2775	3.30E-28	1.49E-26	96	92
GO:0009790	embryo development	63.2024	3.56E-28	1.61E-26	408	288
GO:0002181	cytoplasmic translation	63.1344	3.81E-28	1.72E-26	87	85

GO:0030029	actin filament-based process	63.0502	4.15E-28	1.86E-26	403	285
GO:0045859	regulation of protein kinase activity	62.9783	4.46E-28	2.00E-26	578	383
GO:0090150	establishment of protein localization to membrane	62.9596	4.54E-28	2.03E-26	203	165
GO:1901988	negative regulation of cell cycle phase transition	62.9455	4.60E-28	2.06E-26	222	177
GO:0009199	ribonucleoside triphosphate metabolic process	62.8338	5.15E-28	2.30E-26	125	113
GO:0032434	regulation of proteasomal ubiquitin-dependent protein catabol	62.7192	5.77E-28	2.57E-26	138	122
GO:0016236	macroautophagy	62.4895	7.26E-28	3.23E-26	103	97
GO:0019693	ribose phosphate metabolic process	62.2588	9.15E-28	4.06E-26	325	239
GO:0001701	in utero embryonic development	62.0291	1.15E-27	5.07E-26	327	240
GO:0016032	viral process	61.9098	1.30E-27	5.71E-26	90	87
GO:0043009	chordate embryonic development	61.8812	1.33E-27	5.86E-26	334	244
GO:0140694	non-membrane-bounded organelle assembly	61.8016	1.45E-27	6.33E-26	236	185
GO:0009142	nucleoside triphosphate biosynthetic process	61.524	1.91E-27	8.34E-26	85	83
GO:0009259	ribonucleotide metabolic process	61.3723	2.22E-27	9.67E-26	316	233
GO:0034097	response to cytokine	61.2485	2.51E-27	1.09E-25	596	391
GO:0034504	protein localization to nucleus	61.144	2.79E-27	1.21E-25	172	144
GO:0030162	regulation of proteolysis	61.0965	2.92E-27	1.27E-25	756	477
GO:0043087	regulation of GTPase activity	60.8201	3.86E-27	1.67E-25	344	249
GO:0032956	regulation of actin cytoskeleton organization	60.2186	7.04E-27	3.03E-25	350	252
GO:1901361	organic cyclic compound catabolic process	60.2059	7.13E-27	3.06E-25	338	245
GO:0009144	purine nucleoside triphosphate metabolic process	60.2035	7.14E-27	3.07E-25	128	114
GO:0006888	endoplasmic reticulum to Golgi vesicle-mediated transport	60.1635	7.44E-27	3.18E-25	111	102
GO:0003714	transcription corepressor activity	60.1517	7.52E-27	3.22E-25	193	157
GO:0009792	embryo development ending in birth or egg hatching	59.7121	1.17E-26	4.98E-25	349	251
GO:0009260	ribonucleotide biosynthetic process	59.6957	1.19E-26	5.05E-25	164	138
GO:0098876	vesicle-mediated transport to the plasma membrane	59.6865	1.20E-26	5.08E-25	124	111
GO:0007017	microtubule-based process	59.4919	1.46E-26	6.16E-25	807	502
GO:1903008	organelle disassembly	59.416	1.57E-26	6.63E-25	99	93
GO:0046390	ribose phosphate biosynthetic process	59.276	1.81E-26	7.59E-25	172	143
GO:0043414	macromolecule methylation	59.2318	1.89E-26	7.92E-25	186	152
GO:0051336	regulation of hydrolase activity	59.0345	2.30E-26	9.63E-25	965	584
GO:0051094	positive regulation of developmental process	59.0258	2.32E-26	9.69E-25	1498	855
GO:0008654	phospholipid biosynthetic process	58.961	2.47E-26	1.03E-24	202	162
GO:0016311	dephosphorylation	58.922	2.57E-26	1.07E-24	210	167
GO:0009451	RNA modification	58.91	2.60E-26	1.08E-24	145	125
GO:0072522	purine-containing compound biosynthetic process	58.7483	3.06E-26	1.27E-24	188	153
GO:0042326	negative regulation of phosphorylation	58.6399	3.41E-26	1.41E-24	354	253
GO:0042274	ribosomal small subunit biogenesis	58.6379	3.42E-26	1.41E-24	71	71

GO:0051348	negative regulation of transferase activity	58.5851	3.60E-26	1.48E-24	246	189
GO:0006457	protein folding	58.5352	3.79E-26	1.56E-24	168	140
GO:0009205	purine ribonucleoside triphosphate metabolic process	58.4717	4.04E-26	1.66E-24	119	107
GO:0005525	GTP binding	58.4561	4.10E-26	1.68E-24	356	254
GO:0016482	cytosolic transport	58.3135	4.73E-26	1.93E-24	138	120
GO:0043022	ribosome binding	58.3131	4.73E-26	1.93E-24	112	102
GO:0010628	positive regulation of gene expression	58.0282	6.29E-26	2.56E-24	1253	730
GO:0015931	nucleobase-containing compound transport	57.8211	7.74E-26	3.14E-24	192	155
GO:0006260	DNA replication	57.7885	7.99E-26	3.24E-24	131	115
GO:0032561	guanyl ribonucleotide binding	57.5517	1.01E-25	4.09E-24	380	267
GO:0019001	guanyl nucleotide binding	57.5517	1.01E-25	4.09E-24	380	267
GO:0006644	phospholipid metabolic process	57.5432	1.02E-25	4.12E-24	328	237
GO:0006402	mRNA catabolic process	57.5312	1.03E-25	4.16E-24	121	108
GO:0016741	transferase activity, transferring one-carbon groups	57.5165	1.05E-25	4.22E-24	205	163
GO:1902533	positive regulation of intracellular signal transduction	57.4566	1.11E-25	4.47E-24	1045	623
GO:0032388	positive regulation of intracellular transport	57.2031	1.44E-25	5.75E-24	191	154
GO:0033157	regulation of intracellular protein transport	57.1189	1.56E-25	6.24E-24	228	177
GO:1904951	positive regulation of establishment of protein localization	57.1087	1.58E-25	6.29E-24	358	254
GO:0045787	positive regulation of cell cycle	57.1087	1.58E-25	6.29E-24	358	254
GO:0000226	microtubule cytoskeleton organization	57.0921	1.60E-25	6.38E-24	448	305
GO:0098772	molecular function regulator activity	57.0836	1.62E-25	6.42E-24	2038	1118
GO:0009150	purine ribonucleotide metabolic process	57.0191	1.73E-25	6.84E-24	298	219
GO:0032970	regulation of actin filament-based process	56.9471	1.85E-25	7.32E-24	395	275
GO:0009653	anatomical structure morphogenesis	56.8675	2.01E-25	7.92E-24	1586	895
GO:0071495	cellular response to endogenous stimulus	56.8372	2.07E-25	8.15E-24	702	443
GO:0051656	establishment of organelle localization	56.8349	2.07E-25	8.15E-24	329	237
GO:0009201	ribonucleoside triphosphate biosynthetic process	56.7011	2.37E-25	9.25E-24	79	77
GO:0140101	catalytic activity, acting on a tRNA	56.549	2.76E-25	1.08E-23	113	102
GO:0007264	small GTPase mediated signal transduction	56.3141	3.49E-25	1.35E-23	242	185
GO:0042770	signal transduction in response to DNA damage	56.3047	3.53E-25	1.36E-23	132	115
GO:0009152	purine ribonucleotide biosynthetic process	56.282	3.61E-25	1.39E-23	153	129
GO:1901700	response to oxygen-containing compound	55.9218	5.17E-25	1.99E-23	1397	799
GO:0030036	actin cytoskeleton organization	55.6369	6.87E-25	2.64E-23	355	251
GO:0006164	purine nucleotide biosynthetic process	55.4701	8.12E-25	3.11E-23	180	146
GO:0010975	regulation of neuron projection development	55.3925	8.78E-25	3.36E-23	573	372
GO:0034249	negative regulation of amide metabolic process	55.3568	9.10E-25	3.47E-23	188	151
GO:0042578	phosphoric ester hydrolase activity	55.2062	1.06E-24	4.03E-23	340	242
GO:0006650	glycerophospholipid metabolic process	55.1315	1.14E-24	4.33E-23	250	189

GO:0070848	response to growth factor	54.8509	1.51E-24	5.72E-23	257	193
GO:0110053	regulation of actin filament organization	54.7228	1.71E-24	6.49E-23	279	206
GO:0051049	regulation of transport	54.6229	1.89E-24	7.14E-23	1944	1067
GO:0000075	cell cycle checkpoint signaling	54.554	2.03E-24	7.64E-23	159	132
GO:0008168	methyltransferase activity	54.5101	2.12E-24	7.94E-23	192	153
GO:0120031	plasma membrane bounded cell projection assembly	54.4948	2.15E-24	8.05E-23	362	254
GO:0032204	regulation of telomere maintenance	54.3001	2.62E-24	9.76E-23	96	89
GO:2001234	negative regulation of apoptotic signaling pathway	54.2994	2.62E-24	9.76E-23	251	189
GO:0009145	purine nucleoside triphosphate biosynthetic process	54.2945	2.63E-24	9.79E-23	76	74
GO:0042592	homeostatic process	54.2502	2.75E-24	1.02E-22	1335	765
GO:0097193	intrinsic apoptotic signaling pathway	54.2265	2.82E-24	1.04E-22	178	144
GO:0005085	guanyl-nucleotide exchange factor activity	54.131	3.10E-24	1.15E-22	202	159
GO:0005096	GTPase activator activity	54.0639	3.31E-24	1.22E-22	238	181
GO:0030705	cytoskeleton-dependent intracellular transport	53.9175	3.84E-24	1.42E-22	199	157
GO:0043130	ubiquitin binding	53.8587	4.07E-24	1.50E-22	99	91
GO:0051345	positive regulation of hydrolase activity	53.7758	4.42E-24	1.62E-22	534	349
GO:0007041	lysosomal transport	53.6932	4.80E-24	1.76E-22	122	107
GO:0072659	protein localization to plasma membrane	53.5079	5.78E-24	2.11E-22	214	166
GO:2001242	regulation of intrinsic apoptotic signaling pathway	53.5025	5.81E-24	2.12E-22	193	153
GO:0009206	purine ribonucleoside triphosphate biosynthetic process	53.4931	5.86E-24	2.14E-22	75	73
GO:0140657	ATP-dependent activity	53.4229	6.29E-24	2.29E-22	489	324
GO:0009411	response to UV	53.1631	8.16E-24	2.97E-22	154	128
GO:0032784	regulation of DNA-templated transcription elongation	53.1446	8.31E-24	3.02E-22	87	82
GO:0030031	cell projection assembly	52.9612	9.98E-24	3.62E-22	375	260
GO:0010508	positive regulation of autophagy	52.7381	1.25E-23	4.51E-22	159	131
GO:0016922	nuclear receptor binding	52.6225	1.40E-23	5.05E-22	156	129
GO:0015980	energy derivation by oxidation of organic compounds	52.3063	1.92E-23	6.92E-22	183	146
GO:0034250	positive regulation of amide metabolic process	52.04	2.51E-23	9.00E-22	169	137
GO:0009314	response to radiation	51.9693	2.69E-23	9.64E-22	423	286
GO:0019843	rRNA binding	51.8915	2.91E-23	1.04E-21	73	71
GO:0046486	glycerolipid metabolic process	51.7906	3.22E-23	1.15E-21	326	231
GO:0009060	aerobic respiration	51.7217	3.45E-23	1.23E-21	103	93
GO:1903052	positive regulation of proteolysis involved in protein catabolic p	51.4575	4.49E-23	1.60E-21	128	110
GO:0046474	glycerophospholipid biosynthetic process	51.3417	5.04E-23	1.79E-21	154	127
GO:0060284	regulation of cell development	51.2938	5.29E-23	1.87E-21	956	568
GO:2000045	regulation of G1/S transition of mitotic cell cycle	51.1085	6.37E-23	2.25E-21	162	132
GO:0031123	RNA 3'-end processing	51.0235	6.93E-23	2.45E-21	88	82
GO:0045333	cellular respiration	50.9981	7.11E-23	2.51E-21	115	101

GO:0040029	epigenetic regulation of gene expression	50.9558	7.42E-23	2.61E-21	194	152
GO:0016241	regulation of macroautophagy	50.9047	7.80E-23	2.74E-21	118	103
GO:0051236	establishment of RNA localization	50.8887	7.93E-23	2.78E-21	139	117
GO:0051347	positive regulation of transferase activity	50.8777	8.02E-23	2.81E-21	494	324
GO:0007051	spindle organization	50.7033	9.55E-23	3.34E-21	153	126
GO:0008610	lipid biosynthetic process	50.5864	1.07E-22	3.75E-21	524	340
GO:0060491	regulation of cell projection assembly	50.5612	1.10E-22	3.83E-21	214	164
GO:0030335	positive regulation of cell migration	50.5597	1.10E-22	3.83E-21	589	375
GO:0015631	tubulin binding	50.5564	1.11E-22	3.84E-21	379	260
GO:0016791	phosphatase activity	50.5553	1.11E-22	3.84E-21	254	188
GO:0051222	positive regulation of protein transport	50.4867	1.19E-22	4.10E-21	342	239
GO:0004386	helicase activity	50.48	1.19E-22	4.12E-21	147	122
GO:0044255	cellular lipid metabolic process	50.4519	1.23E-22	4.23E-21	904	540
GO:0010970	transport along microtubule	50.1796	1.61E-22	5.53E-21	166	134
GO:0000956	nuclear-transcribed mRNA catabolic process	50.1434	1.67E-22	5.72E-21	94	86
GO:1903432	regulation of TORC1 signaling	49.7931	2.37E-22	8.08E-21	90	83
GO:0000287	magnesium ion binding	49.7402	2.50E-22	8.51E-21	210	161
GO:1901800	positive regulation of proteasomal protein catabolic process	49.7107	2.58E-22	8.75E-21	110	97
GO:0001933	negative regulation of protein phosphorylation	49.6747	2.67E-22	9.06E-21	324	228
GO:0050658	RNA transport	49.5734	2.96E-22	9.99E-21	137	115
GO:0050657	nucleic acid transport	49.5734	2.96E-22	9.99E-21	137	115
GO:0017148	negative regulation of translation	49.5623	2.99E-22	1.01E-20	165	133
GO:0045017	glycerolipid biosynthetic process	49.4979	3.19E-22	1.07E-20	181	143
GO:2000147	positive regulation of cell motility	49.401	3.51E-22	1.18E-20	616	388
GO:0010562	positive regulation of phosphorus metabolic process	49.2828	3.95E-22	1.32E-20	855	513
GO:0045937	positive regulation of phosphate metabolic process	49.2828	3.95E-22	1.32E-20	855	513
GO:0034243	regulation of transcription elongation by RNA polymerase II	49.2496	4.08E-22	1.37E-20	74	71
GO:0045930	negative regulation of mitotic cell cycle	49.0968	4.76E-22	1.59E-20	219	166
GO:0040017	positive regulation of locomotion	49.0016	5.23E-22	1.75E-20	632	396
GO:0045727	positive regulation of translation	48.9971	5.26E-22	1.75E-20	139	116
GO:0120032	regulation of plasma membrane bounded cell projection assem	48.8666	5.99E-22	1.99E-20	211	161
GO:1901701	cellular response to oxygen-containing compound	48.7821	6.52E-22	2.17E-20	905	538
GO:0006754	ATP biosynthetic process	48.4215	9.35E-22	3.10E-20	64	63
GO:0046034	ATP metabolic process	48.3513	1.00E-21	3.32E-20	95	86
GO:0022604	regulation of cell morphogenesis	48.3397	1.01E-21	3.35E-20	274	198
GO:0048513	animal organ development	48.3122	1.04E-21	3.43E-20	1447	810
GO:0051168	nuclear export	48.2636	1.09E-21	3.59E-20	135	113
GO:1901699	cellular response to nitrogen compound	48.242	1.12E-21	3.67E-20	419	280

GO:0010212	response to ionizing radiation	48.1381	1.24E-21	4.06E-20	129	109
GO:1903322	positive regulation of protein modification by small protein con	47.9107	1.56E-21	5.09E-20	143	118
GO:0051276	chromosome organization	47.6915	1.94E-21	6.33E-20	343	237
GO:0019207	kinase regulator activity	47.6288	2.07E-21	6.73E-20	263	191
GO:0045597	positive regulation of cell differentiation	47.6008	2.12E-21	6.91E-20	979	574
GO:0006892	post-Golgi vesicle-mediated transport	47.5523	2.23E-21	7.25E-20	87	80
GO:0043393	regulation of protein binding	47.4462	2.48E-21	8.05E-20	226	169
GO:0006897	endocytosis	47.369	2.68E-21	8.68E-20	437	289
GO:0062197	cellular response to chemical stress	47.2641	2.97E-21	9.62E-20	238	176
GO:1901991	negative regulation of mitotic cell cycle phase transition	47.1082	3.48E-21	1.12E-19	161	129
GO:0071345	cellular response to cytokine stimulus	47.0774	3.59E-21	1.15E-19	465	304
GO:0008757	S-adenosylmethionine-dependent methyltransferase activity	47.0517	3.68E-21	1.18E-19	150	122
GO:0071363	cellular response to growth factor stimulus	46.974	3.98E-21	1.28E-19	235	174
GO:0019902	phosphatase binding	46.9333	4.14E-21	1.33E-19	230	171
GO:0032271	regulation of protein polymerization	46.9219	4.19E-21	1.34E-19	205	156
GO:0035091	phosphatidylinositol binding	46.6549	5.47E-21	1.75E-19	292	207
GO:0030155	regulation of cell adhesion	46.5337	6.18E-21	1.97E-19	828	495
GO:0051093	negative regulation of developmental process	46.5185	6.27E-21	2.00E-19	1010	588
GO:0022900	electron transport chain	46.4637	6.62E-21	2.11E-19	99	88
GO:0007167	enzyme-linked receptor protein signaling pathway	46.4245	6.89E-21	2.19E-19	576	363
GO:1902806	regulation of cell cycle G1/S phase transition	46.293	7.86E-21	2.49E-19	186	144
GO:2000060	positive regulation of ubiquitin-dependent protein catabolic prc	46.2037	8.59E-21	2.71E-19	105	92
GO:0000049	tRNA binding	46.1067	9.47E-21	2.98E-19	70	67
GO:1990778	protein localization to cell periphery	46.0039	1.05E-20	3.30E-19	267	192
GO:0010257	NADH dehydrogenase complex assembly	45.9873	1.07E-20	3.34E-19	61	60
GO:0032981	mitochondrial respiratory chain complex I assembly	45.9873	1.07E-20	3.34E-19	61	60
GO:0031334	positive regulation of protein-containing complex assembly	45.7885	1.30E-20	4.07E-19	208	157
GO:1901874	negative regulation of post-translational protein modification	45.7521	1.35E-20	4.22E-19	98	87
GO:0006400	tRNA modification	45.7355	1.37E-20	4.28E-19	81	75
GO:0018105	peptidyl-serine phosphorylation	45.7171	1.40E-20	4.36E-19	185	143
GO:0045739	positive regulation of DNA repair	45.6618	1.48E-20	4.60E-19	131	109
GO:0000077	DNA damage checkpoint signaling	45.611	1.55E-20	4.82E-19	101	89
GO:0048869	cellular developmental process	45.4593	1.81E-20	5.60E-19	3090	1594
GO:2001252	positive regulation of chromosome organization	45.4336	1.86E-20	5.74E-19	107	93
GO:0071900	regulation of protein serine/threonine kinase activity	45.3813	1.95E-20	6.04E-19	338	232
GO:0018209	peptidyl-serine modification	45.2776	2.17E-20	6.69E-19	197	150
GO:0051090	regulation of DNA-binding transcription factor activity	45.1852	2.38E-20	7.31E-19	434	285
GO:0003924	GTPase activity	45.0215	2.80E-20	8.59E-19	298	209

GO:0007006	mitochondrial membrane organization	44.7441	3.70E-20	1.13E-18	106	92
GO:0006629	lipid metabolic process	44.7408	3.71E-20	1.13E-18	1211	686
GO:0071496	cellular response to external stimulus	44.705	3.84E-20	1.17E-18	288	203
GO:0033673	negative regulation of kinase activity	44.6959	3.88E-20	1.18E-18	211	158
GO:0007265	Ras protein signal transduction	44.6591	4.03E-20	1.23E-18	178	138
GO:0005198	structural molecule activity	44.5469	4.50E-20	1.37E-18	664	407
GO:0060627	regulation of vesicle-mediated transport	44.4741	4.84E-20	1.47E-18	609	378
GO:0042327	positive regulation of phosphorylation	44.4192	5.12E-20	1.55E-18	774	464
GO:0042393	histone binding	44.386	5.29E-20	1.60E-18	285	201
GO:0003697	single-stranded DNA binding	44.1889	6.44E-20	1.94E-18	123	103
GO:0046488	phosphatidylinositol metabolic process	44.1019	7.03E-20	2.12E-18	137	112
GO:0031570	DNA integrity checkpoint signaling	44.0243	7.59E-20	2.28E-18	108	93
GO:0022904	respiratory electron transport chain	43.9913	7.85E-20	2.36E-18	89	80
GO:0010821	regulation of mitochondrion organization	43.9456	8.22E-20	2.47E-18	145	117
GO:0003779	actin binding	43.8739	8.83E-20	2.65E-18	444	289
GO:1905037	autophagosome organization	43.4839	1.30E-19	3.89E-18	78	72
GO:0043543	protein acylation	43.404	1.41E-19	4.21E-18	116	98
GO:2001243	negative regulation of intrinsic apoptotic signaling pathway	43.343	1.50E-19	4.47E-18	110	94
GO:0032436	positive regulation of proteasomal ubiquitin-dependent proteir	43.2689	1.62E-19	4.81E-18	88	79
GO:0104004	cellular response to environmental stimulus	43.2632	1.63E-19	4.82E-18	278	196
GO:0071214	cellular response to abiotic stimulus	43.2632	1.63E-19	4.82E-18	278	196
GO:0032535	regulation of cellular component size	43.2581	1.63E-19	4.84E-18	285	200
GO:0001098	basal transcription machinery binding	43.1155	1.88E-19	5.56E-18	62	60
GO:0001099	basal RNA polymerase II transcription machinery binding	43.1155	1.88E-19	5.56E-18	62	60
GO:0034599	cellular response to oxidative stress	43.0738	1.96E-19	5.79E-18	188	143
GO:0006302	double-strand break repair	43.0494	2.01E-19	5.93E-18	193	146
GO:0042776	proton motive force-driven mitochondrial ATP synthesis	42.9202	2.29E-19	6.73E-18	52	52
GO:0015986	proton motive force-driven ATP synthesis	42.9202	2.29E-19	6.73E-18	52	52
GO:0032989	cellular component morphogenesis	42.7427	2.74E-19	8.02E-18	339	230
GO:0032990	cell part morphogenesis	42.7427	2.74E-19	8.02E-18	339	230
GO:0010810	regulation of cell-substrate adhesion	42.2398	4.52E-19	1.32E-17	226	165
GO:0050684	regulation of mRNA processing	42.2193	4.62E-19	1.35E-17	134	109
GO:0006470	protein dephosphorylation	42.0707	5.36E-19	1.56E-17	150	119
GO:0045931	positive regulation of mitotic cell cycle	41.9335	6.15E-19	1.79E-17	128	105
GO:0048646	anatomical structure formation involved in morphogenesis	41.8431	6.73E-19	1.95E-17	846	497
GO:0051240	positive regulation of multicellular organismal process	41.7018	7.75E-19	2.25E-17	1856	996
GO:0090068	positive regulation of cell cycle process	41.6575	8.10E-19	2.34E-17	270	190
GO:0071417	cellular response to organonitrogen compound	41.5196	9.30E-19	2.68E-17	381	252

GO:1903321	negative regulation of protein modification by small protein co	41.5109	9.38E-19	2.70E-17	92	81
GO:0090316	positive regulation of intracellular protein transport	41.4712	9.76E-19	2.81E-17	149	118
GO:0016887	ATP hydrolysis activity	41.337	1.12E-18	3.21E-17	274	192
GO:0051056	regulation of small GTPase mediated signal transduction	41.299	1.16E-18	3.33E-17	229	166
GO:0019725	cellular homeostasis	41.261	1.20E-18	3.45E-17	644	392
GO:0005975	carbohydrate metabolic process	41.2306	1.24E-18	3.56E-17	409	267
GO:0061013	regulation of mRNA catabolic process	41.1632	1.33E-18	3.80E-17	159	124
GO:0051099	positive regulation of binding	41.1428	1.35E-18	3.87E-17	197	147
GO:1902749	regulation of cell cycle G2/M phase transition	40.8471	1.82E-18	5.20E-17	115	96
GO:0140104	molecular carrier activity	40.8091	1.89E-18	5.39E-17	91	80
GO:1902850	microtubule cytoskeleton organization involved in mitosis	40.6704	2.17E-18	6.17E-17	126	103
GO:0030307	positive regulation of cell growth	40.6034	2.32E-18	6.59E-17	196	146
GO:0032456	endocytic recycling	40.4969	2.58E-18	7.32E-17	74	68
GO:1900180	regulation of protein localization to nucleus	40.4783	2.63E-18	7.45E-17	163	126
GO:0030154	cell differentiation	39.8856	4.76E-18	1.34E-16	2505	1302
GO:0070585	protein localization to mitochondrion	39.8844	4.77E-18	1.34E-16	80	72
GO:0031669	cellular response to nutrient levels	39.7714	5.34E-18	1.50E-16	226	163
GO:0032984	protein-containing complex disassembly	39.7333	5.55E-18	1.55E-16	130	105
GO:0007093	mitotic cell cycle checkpoint signaling	39.7333	5.55E-18	1.55E-16	130	105
GO:0042147	retrograde transport, endosome to Golgi	39.6771	5.87E-18	1.64E-16	83	74
GO:0016779	nucleotidyltransferase activity	39.6673	5.93E-18	1.65E-16	138	110
GO:0051170	import into nucleus	39.654	6.00E-18	1.67E-16	116	96
GO:0140241	translation at synapse	39.6136	6.25E-18	1.74E-16	48	48
GO:0140242	translation at postsynapse	39.6136	6.25E-18	1.74E-16	48	48
GO:0019887	protein kinase regulator activity	39.5745	6.50E-18	1.81E-16	228	164
GO:0031330	negative regulation of cellular catabolic process	39.4974	7.02E-18	1.95E-16	189	141
GO:0060260	regulation of transcription initiation by RNA polymerase II	39.4201	7.59E-18	2.10E-16	76	69
GO:0006473	protein acetylation	39.4201	7.59E-18	2.10E-16	76	69
GO:0065004	protein-DNA complex assembly	39.2641	8.87E-18	2.45E-16	140	111
GO:0045182	translation regulator activity	39.2641	8.87E-18	2.45E-16	140	111
GO:0006446	regulation of translational initiation	39.1867	9.58E-18	2.64E-16	65	61
GO:0032786	positive regulation of DNA-templated transcription, elongation	39.1867	9.58E-18	2.64E-16	65	61
GO:0031668	cellular response to extracellular stimulus	39.0953	1.05E-17	2.89E-16	253	178
GO:0140296	general transcription initiation factor binding	39.0761	1.07E-17	2.94E-16	61	58
GO:0002682	regulation of immune system process	39.051	1.10E-17	3.01E-16	1590	861
GO:2000142	regulation of DNA-templated transcription initiation	38.8161	1.39E-17	3.80E-16	85	75
GO:0140236	translation at presynapse	38.7871	1.43E-17	3.90E-16	47	47
GO:0006469	negative regulation of protein kinase activity	38.708	1.55E-17	4.22E-16	190	141

GO:0007169	transmembrane receptor protein tyrosine kinase signaling patl	38.5467	1.82E-17	4.94E-16	398	258
GO:0043122	regulation of canonical NF-kappaB signal transduction	38.4504	2.00E-17	5.43E-16	209	152
GO:0001934	positive regulation of protein phosphorylation	38.3293	2.26E-17	6.13E-16	720	427
GO:0005543	phospholipid binding	38.3106	2.30E-17	6.23E-16	488	306
GO:0070063	RNA polymerase binding	38.2989	2.33E-17	6.30E-16	60	57
GO:0000045	autophagosome assembly	38.2692	2.40E-17	6.48E-16	71	65
GO:0051028	mRNA transport	38.2596	2.42E-17	6.54E-16	111	92
GO:0031397	negative regulation of protein ubiquitination	38.2504	2.44E-17	6.59E-16	81	72
GO:0051494	negative regulation of cytoskeleton organization	38.114	2.80E-17	7.52E-16	169	128
GO:0051225	spindle assembly	38.1123	2.81E-17	7.52E-16	84	74
GO:0046777	protein autophosphorylation	37.9939	3.16E-17	8.46E-16	196	144
GO:2000144	positive regulation of DNA-templated transcription initiation	37.9577	3.27E-17	8.75E-16	74	67
GO:0061912	selective autophagy	37.919	3.40E-17	9.07E-16	67	62
GO:0060261	positive regulation of transcription initiation by RNA polymeras	37.919	3.40E-17	9.07E-16	67	62
GO:0044770	cell cycle phase transition	37.7885	3.88E-17	1.03E-15	148	115
GO:0016874	ligase activity	37.6593	4.41E-17	1.17E-15	132	105
GO:0070897	transcription preinitiation complex assembly	37.5655	4.85E-17	1.29E-15	55	53
GO:0071478	cellular response to radiation	37.5603	4.87E-17	1.29E-15	168	127
GO:1902904	negative regulation of supramolecular fiber organization	37.5565	4.89E-17	1.30E-15	173	130
GO:0032206	positive regulation of telomere maintenance	37.5292	5.03E-17	1.33E-15	70	64
GO:0072655	establishment of protein localization to mitochondrion	37.5292	5.03E-17	1.33E-15	70	64
GO:0070085	glycosylation	37.5276	5.03E-17	1.33E-15	212	153
GO:0045185	maintenance of protein location	37.4216	5.60E-17	1.48E-15	104	87
GO:0036503	ERAD pathway	37.2725	6.50E-17	1.71E-15	89	77
GO:0046822	regulation of nucleocytoplasmic transport	37.2625	6.56E-17	1.72E-15	134	106
GO:0006661	phosphatidylinositol biosynthetic process	37.2618	6.57E-17	1.72E-15	95	81
GO:0007166	cell surface receptor signaling pathway	37.2057	6.95E-17	1.82E-15	1816	966
GO:0048878	chemical homeostasis	37.1525	7.33E-17	1.92E-15	891	512
GO:1902117	positive regulation of organelle assembly	36.588	1.29E-16	3.36E-15	88	76
GO:0043487	regulation of RNA stability	36.5537	1.33E-16	3.48E-15	151	116
GO:0010632	regulation of epithelial cell migration	36.5372	1.36E-16	3.53E-15	243	170
GO:1903828	negative regulation of protein localization	36.5372	1.36E-16	3.53E-15	243	170
GO:0006606	protein import into nucleus	36.4679	1.45E-16	3.78E-15	111	91
GO:0043547	positive regulation of GTPase activity	36.4082	1.54E-16	4.00E-15	252	175
GO:0006366	transcription by RNA polymerase II	36.3646	1.61E-16	4.17E-15	268	184
GO:0007015	actin filament organization	36.2339	1.84E-16	4.74E-15	270	185
GO:0060606	tube closure	36.2221	1.86E-16	4.79E-15	105	87
GO:0032886	regulation of microtubule-based process	36.1721	1.95E-16	5.03E-15	263	181

GO:1901989	positive regulation of cell cycle phase transition	36.14	2.02E-16	5.19E-15	116	94
GO:0140030	modification-dependent protein binding	36.0624	2.18E-16	5.59E-15	228	161
GO:0031333	negative regulation of protein-containing complex assembly	35.9862	2.35E-16	6.02E-15	150	115
GO:0032968	positive regulation of transcription elongation by RNA polymera	35.9858	2.35E-16	6.02E-15	53	51
GO:0070507	regulation of microtubule cytoskeleton organization	35.9151	2.53E-16	6.44E-15	160	121
GO:0097435	supramolecular fiber organization	35.7804	2.89E-16	7.35E-15	538	329
GO:0030111	regulation of Wnt signaling pathway	35.7799	2.89E-16	7.35E-15	323	214
GO:0042594	response to starvation	35.7045	3.12E-16	7.92E-15	206	148
GO:0070646	protein modification by small protein removal	35.6914	3.16E-16	8.01E-15	118	95
GO:0040007	growth	35.6771	3.20E-16	8.12E-15	451	283
GO:0008094	ATP-dependent activity, acting on DNA	35.52	3.75E-16	9.49E-15	115	93
GO:0045927	positive regulation of growth	35.5102	3.79E-16	9.57E-15	309	206
GO:0051983	regulation of chromosome segregation	35.4794	3.90E-16	9.86E-15	131	103
GO:0001843	neural tube closure	35.4732	3.93E-16	9.91E-15	101	84
GO:0031593	polyubiquitin modification-dependent protein binding	35.3757	4.33E-16	1.09E-14	60	56
GO:0019903	protein phosphatase binding	35.2381	4.97E-16	1.25E-14	181	133
GO:0004518	nuclease activity	35.2028	5.15E-16	1.29E-14	205	147
GO:0043565	sequence-specific DNA binding	34.9874	6.38E-16	1.60E-14	1568	841
GO:0051495	positive regulation of cytoskeleton organization	34.9781	6.44E-16	1.62E-14	195	141
GO:0006310	DNA recombination	34.9413	6.69E-16	1.68E-14	221	156
GO:1903313	positive regulation of mRNA metabolic process	34.9403	6.69E-16	1.68E-14	103	85
GO:0043488	regulation of mRNA stability	34.7613	8.00E-16	2.00E-14	135	105
GO:0048589	developmental growth	34.6214	9.21E-16	2.29E-14	446	279
GO:0016239	positive regulation of macroautophagy	34.6178	9.24E-16	2.30E-14	79	69
GO:0006486	protein glycosylation	34.5766	9.63E-16	2.39E-14	187	136
GO:0043413	macromolecule glycosylation	34.5766	9.63E-16	2.39E-14	187	136
GO:0006839	mitochondrial transport	34.5645	9.75E-16	2.42E-14	82	71
GO:0031398	positive regulation of protein ubiquitination	34.4663	1.08E-15	2.66E-14	116	93
GO:0006414	translational elongation	34.4092	1.14E-15	2.81E-14	51	49
GO:0016491	oxidoreductase activity	34.3668	1.19E-15	2.93E-14	756	439
GO:2000116	regulation of cysteine-type endopeptidase activity	34.3225	1.24E-15	3.06E-14	236	164
GO:0010389	regulation of G2/M transition of mitotic cell cycle	34.3025	1.27E-15	3.12E-14	102	84
GO:0016706	2-oxoglutarate-dependent dioxygenase activity	34.1792	1.43E-15	3.53E-14	62	57
GO:0045814	negative regulation of gene expression, epigenetic	34.0813	1.58E-15	3.88E-14	96	80
GO:0048284	organelle fusion	34.0783	1.58E-15	3.89E-14	126	99
GO:1901992	positive regulation of mitotic cell cycle phase transition	33.996	1.72E-15	4.22E-14	93	78
GO:0006275	regulation of DNA replication	33.8613	1.97E-15	4.82E-14	123	97
GO:0042177	negative regulation of protein catabolic process	33.8568	1.98E-15	4.83E-14	115	92

GO:0099111	microtubule-based transport	33.8458	2.00E-15	4.88E-14	207	147
GO:0004721	phosphoprotein phosphatase activity	33.7192	2.27E-15	5.53E-14	156	117
GO:0051235	maintenance of location	33.6729	2.38E-15	5.79E-14	178	130
GO:0048858	cell projection morphogenesis	33.5328	2.73E-15	6.63E-14	311	205
GO:1905475	regulation of protein localization to membrane	33.5025	2.82E-15	6.83E-14	211	149
GO:0048024	regulation of mRNA splicing, via spliceosome	33.4967	2.84E-15	6.85E-14	109	88
GO:0090079	translation regulator activity, nucleic acid binding	33.4967	2.84E-15	6.85E-14	109	88
GO:0019752	carboxylic acid metabolic process	33.3034	3.44E-15	8.30E-14	823	471
GO:0044772	mitotic cell cycle phase transition	33.2752	3.54E-15	8.53E-14	135	104
GO:0008064	regulation of actin polymerization or depolymerization	33.2448	3.65E-15	8.79E-14	165	122
GO:0004527	exonuclease activity	33.2324	3.69E-15	8.89E-14	77	67
GO:0000302	response to reactive oxygen species	33.2019	3.81E-15	9.15E-14	160	119
GO:0031072	heat shock protein binding	33.1672	3.94E-15	9.46E-14	150	113
GO:0033674	positive regulation of kinase activity	33.0998	4.22E-15	1.01E-13	412	259
GO:0008017	microtubule binding	32.97	4.80E-15	1.15E-13	265	179
GO:0120039	plasma membrane bounded cell projection morphogenesis	32.9659	4.82E-15	1.15E-13	303	200
GO:0046578	regulation of Ras protein signal transduction	32.9014	5.14E-15	1.23E-13	198	141
GO:0035148	tube formation	32.8962	5.17E-15	1.23E-13	152	114
GO:0018394	peptidyl-lysine acetylation	32.8853	5.23E-15	1.25E-13	53	50
GO:0051123	RNA polymerase II preinitiation complex assembly	32.8358	5.49E-15	1.31E-13	49	47
GO:0032200	telomere organization	32.7886	5.76E-15	1.37E-13	94	78
GO:0000723	telomere maintenance	32.7886	5.76E-15	1.37E-13	94	78
GO:0010976	positive regulation of neuron projection development	32.7837	5.78E-15	1.37E-13	235	162
GO:0045596	negative regulation of cell differentiation	32.7325	6.09E-15	1.44E-13	720	418
GO:0051604	protein maturation	32.7288	6.11E-15	1.45E-13	298	197
GO:0007032	endosome organization	32.6927	6.33E-15	1.50E-13	60	55
GO:0007052	mitotic spindle organization	32.6905	6.35E-15	1.50E-13	91	76
GO:0032006	regulation of TOR signaling	32.6338	6.72E-15	1.59E-13	154	115
GO:0030099	myeloid cell differentiation	32.5851	7.05E-15	1.66E-13	246	168
GO:0030433	ubiquitin-dependent ERAD pathway	32.5425	7.36E-15	1.73E-13	76	66
GO:0002039	p53 binding	32.5425	7.36E-15	1.73E-13	76	66
GO:1905897	regulation of response to endoplasmic reticulum stress	32.515	7.57E-15	1.78E-13	79	68
GO:0072384	organelle transport along microtubule	32.515	7.57E-15	1.78E-13	79	68
GO:0030832	regulation of actin filament length	32.2561	9.80E-15	2.30E-13	168	123
GO:0006790	sulfur compound metabolic process	32.2229	1.01E-14	2.37E-13	279	186
GO:0060271	cilium assembly	32.201	1.04E-14	2.42E-13	261	176
GO:0016073	snRNA metabolic process	32.0504	1.20E-14	2.81E-13	48	46
GO:0006261	DNA-templated DNA replication	32.0504	1.20E-14	2.81E-13	48	46

GO:0006997	nucleus organization	32.041	1.22E-14	2.83E-13	90	75
GO:0000281	mitotic cytokinesis	31.9518	1.33E-14	3.09E-13	59	54
GO:0001510	RNA methylation	31.9111	1.38E-14	3.22E-13	72	63
GO:0048812	neuron projection morphogenesis	31.8912	1.41E-14	3.28E-13	296	195
GO:0032868	response to insulin	31.8302	1.50E-14	3.48E-13	135	103
GO:0045926	negative regulation of growth	31.7619	1.61E-14	3.72E-13	260	175
GO:2000146	negative regulation of cell motility	31.7266	1.66E-14	3.85E-13	322	209
GO:0009991	response to extracellular stimulus	31.7226	1.67E-14	3.86E-13	404	253
GO:0031667	response to nutrient levels	31.6561	1.79E-14	4.12E-13	376	238
GO:0090174	organelle membrane fusion	31.6311	1.83E-14	4.22E-13	95	78
GO:0043436	oxoacid metabolic process	31.6296	1.83E-14	4.23E-13	843	478
GO:0043434	response to peptide hormone	31.5636	1.96E-14	4.51E-13	246	167
GO:0030100	regulation of endocytosis	31.5502	1.99E-14	4.57E-13	339	218
GO:0040013	negative regulation of locomotion	31.4837	2.12E-14	4.88E-13	354	226
GO:0008276	protein methyltransferase activity	31.3937	2.32E-14	5.32E-13	89	74
GO:0043086	negative regulation of catalytic activity	31.3835	2.35E-14	5.37E-13	632	371
GO:0006082	organic acid metabolic process	31.3601	2.40E-14	5.49E-13	850	481
GO:1902905	positive regulation of supramolecular fiber organization	31.3365	2.46E-14	5.62E-13	183	131
GO:0003690	double-stranded DNA binding	31.2616	2.65E-14	6.05E-13	1566	831
GO:2001251	negative regulation of chromosome organization	31.2288	2.74E-14	6.25E-13	83	70
GO:1901652	response to peptide	31.0733	3.20E-14	7.28E-13	316	205
GO:0001952	regulation of cell-matrix adhesion	31.0649	3.23E-14	7.34E-13	126	97
GO:0001221	transcription coregulator binding	30.9728	3.54E-14	8.04E-13	141	106
GO:0019646	aerobic electron transport chain	30.9342	3.68E-14	8.34E-13	61	55
GO:0043408	regulation of MAPK cascade	30.9327	3.68E-14	8.35E-13	734	422
GO:0060828	regulation of canonical Wnt signaling pathway	30.8887	3.85E-14	8.71E-13	249	168
GO:0065002	intracellular protein transmembrane transport	30.8423	4.03E-14	9.12E-13	54	50
GO:0017124	SH3 domain binding	30.7501	4.42E-14	9.99E-13	128	98
GO:0003730	mRNA 3'-UTR binding	30.6365	4.95E-14	1.12E-12	107	85
GO:0045860	positive regulation of protein kinase activity	30.5965	5.15E-14	1.16E-12	356	226
GO:0008289	lipid binding	30.5914	5.18E-14	1.16E-12	883	496
GO:1902459	positive regulation of stem cell population maintenance	30.5814	5.23E-14	1.17E-12	50	47
GO:0006475	internal protein amino acid acetylation	30.5814	5.23E-14	1.17E-12	50	47
GO:0018393	internal peptidyl-lysine acetylation	30.5814	5.23E-14	1.17E-12	50	47
GO:0043523	regulation of neuron apoptotic process	30.4958	5.70E-14	1.28E-12	308	200
GO:0044782	cilium organization	30.405	6.24E-14	1.40E-12	288	189
GO:0044283	small molecule biosynthetic process	30.3988	6.28E-14	1.40E-12	430	265
GO:0006906	vesicle fusion	30.368	6.48E-14	1.45E-12	93	76

GO:0009267	cellular response to starvation	30.3432	6.64E-14	1.48E-12	181	129
GO:0051648	vesicle localization	30.336	6.69E-14	1.49E-12	117	91
GO:0043281	regulation of cysteine-type endopeptidase activity involved in	30.3299	6.73E-14	1.50E-12	209	145
GO:0048638	regulation of developmental growth	30.3107	6.86E-14	1.53E-12	398	248
GO:0044403	biological process involved in symbiotic interaction	30.2958	6.96E-14	1.55E-12	195	137
GO:0051650	establishment of vesicle localization	30.2481	7.30E-14	1.62E-12	101	81
GO:0000910	cytokinesis	30.1057	8.42E-14	1.87E-12	87	72
GO:0061640	cytoskeleton-dependent cytokinesis	29.9995	9.36E-14	2.07E-12	84	70
GO:0043244	regulation of protein-containing complex disassembly	29.8745	1.06E-13	2.35E-12	134	101
GO:0044773	mitotic DNA damage checkpoint signaling	29.8488	1.09E-13	2.40E-12	78	66
GO:0150116	regulation of cell-substrate junction organization	29.8313	1.11E-13	2.43E-12	69	60
GO:0051540	metal cluster binding	29.8313	1.11E-13	2.43E-12	69	60
GO:0051536	iron-sulfur cluster binding	29.8313	1.11E-13	2.43E-12	69	60
GO:0035966	response to topologically incorrect protein	29.8032	1.14E-13	2.50E-12	72	62
GO:1904375	regulation of protein localization to cell periphery	29.7991	1.14E-13	2.51E-12	156	114
GO:0034644	cellular response to UV	29.7399	1.21E-13	2.66E-12	92	75
GO:0044088	regulation of vacuole organization	29.7383	1.22E-13	2.66E-12	56	51
GO:0051241	negative regulation of multicellular organismal process	29.6828	1.29E-13	2.81E-12	1243	671
GO:2000779	regulation of double-strand break repair	29.6021	1.39E-13	3.04E-12	131	99
GO:0016477	cell migration	29.3639	1.77E-13	3.85E-12	910	507
GO:0016747	acyltransferase activity, transferring groups other than amino-a	29.3633	1.77E-13	3.85E-12	232	157
GO:0045785	positive regulation of cell adhesion	29.355	1.78E-13	3.87E-12	529	315
GO:0061726	mitochondrion disassembly	29.3423	1.81E-13	3.92E-12	52	48
GO:0000422	autophagy of mitochondrion	29.3423	1.81E-13	3.92E-12	52	48
GO:0005996	monosaccharide metabolic process	29.3395	1.81E-13	3.92E-12	193	135
GO:0051050	positive regulation of transport	29.2704	1.94E-13	4.20E-12	1083	592
GO:0030336	negative regulation of cell migration	29.1546	2.18E-13	4.70E-12	309	199
GO:1903051	negative regulation of proteolysis involved in protein catabolic	29.1237	2.25E-13	4.84E-12	71	61
GO:0043433	negative regulation of DNA-binding transcription factor activity	29.0918	2.32E-13	4.99E-12	169	121
GO:0043175	RNA polymerase core enzyme binding	29.0445	2.43E-13	5.23E-12	40	39
GO:0006479	protein methylation	28.9634	2.64E-13	5.65E-12	88	72
GO:0008213	protein alkylation	28.9634	2.64E-13	5.65E-12	88	72
GO:0019783	ubiquitin-like protein peptidase activity	28.939	2.70E-13	5.79E-12	112	87
GO:0008234	cysteine-type peptidase activity	28.9072	2.79E-13	5.97E-12	171	122
GO:0090287	regulation of cellular response to growth factor stimulus	28.8865	2.85E-13	6.09E-12	317	203
GO:0050767	regulation of neurogenesis	28.8775	2.88E-13	6.14E-12	482	290
GO:0030490	maturation of SSU-rRNA	28.873	2.89E-13	6.15E-12	35	35
GO:0046982	protein heterodimerization activity	28.8294	3.02E-13	6.42E-12	306	197

GO:2000278	regulation of DNA biosynthetic process	28.7786	3.17E-13	6.74E-12	127	96
GO:0006352	DNA-templated transcription initiation	28.7656	3.22E-13	6.83E-12	58	52
GO:0030308	negative regulation of cell growth	28.6107	3.75E-13	7.95E-12	203	140
GO:0070302	regulation of stress-activated protein kinase signaling cascade	28.6107	3.75E-13	7.95E-12	203	140
GO:0051053	negative regulation of DNA metabolic process	28.6101	3.76E-13	7.95E-12	144	106
GO:0030968	endoplasmic reticulum unfolded protein response	28.5946	3.82E-13	8.06E-12	51	47
GO:0051100	negative regulation of binding	28.594	3.82E-13	8.06E-12	168	120
GO:0031507	heterochromatin formation	28.5051	4.17E-13	8.80E-12	64	56
GO:0032507	maintenance of protein location in cell	28.4464	4.42E-13	9.31E-12	70	60
GO:0071479	cellular response to ionizing radiation	28.4464	4.42E-13	9.31E-12	70	60
GO:0072331	signal transduction by p53 class mediator	28.1915	5.71E-13	1.20E-11	84	69
GO:0016573	histone acetylation	28.1378	6.02E-13	1.27E-11	43	41
GO:0010950	positive regulation of endopeptidase activity	28.0985	6.27E-13	1.32E-11	167	119
GO:0010720	positive regulation of cell development	27.9337	7.39E-13	1.55E-11	545	321
GO:2001235	positive regulation of apoptotic signaling pathway	27.8989	7.65E-13	1.60E-11	176	124
GO:0051082	unfolded protein binding	27.8704	7.87E-13	1.65E-11	89	72
GO:2000036	regulation of stem cell population maintenance	27.869	7.88E-13	1.65E-11	75	63
GO:0030833	regulation of actin filament polymerization	27.8614	7.94E-13	1.66E-11	145	106
GO:0009615	response to virus	27.8514	8.02E-13	1.67E-11	310	198
GO:0071897	DNA biosynthetic process	27.7715	8.69E-13	1.81E-11	69	59
GO:0070936	protein K48-linked ubiquitination	27.7706	8.70E-13	1.81E-11	66	57
GO:0016853	isomerase activity	27.7482	8.89E-13	1.85E-11	171	121
GO:0007059	chromosome segregation	27.7331	9.03E-13	1.87E-11	135	100
GO:0071375	cellular response to peptide hormone stimulus	27.7331	9.03E-13	1.87E-11	135	100
GO:0006497	protein lipidation	27.7075	9.26E-13	1.92E-11	86	70
GO:0032869	cellular response to insulin stimulus	27.7075	9.26E-13	1.92E-11	86	70
GO:1900182	positive regulation of protein localization to nucleus	27.6601	9.71E-13	2.01E-11	102	80
GO:0050792	regulation of viral process	27.5803	1.05E-12	2.17E-11	173	122
GO:0008630	intrinsic apoptotic signaling pathway in response to DNA dama	27.5584	1.08E-12	2.22E-11	83	68
GO:0070972	protein localization to endoplasmic reticulum	27.5402	1.10E-12	2.26E-11	53	48
GO:0030374	nuclear receptor coactivator activity	27.5402	1.10E-12	2.26E-11	53	48
GO:0110020	regulation of actomyosin structure organization	27.4639	1.18E-12	2.43E-11	112	86
GO:0051960	regulation of nervous system development	27.3954	1.27E-12	2.60E-11	582	339
GO:0006890	retrograde vesicle-mediated transport, Golgi to endoplasmic re	27.3585	1.31E-12	2.69E-11	42	40
GO:0035601	protein deacylation	27.3585	1.31E-12	2.69E-11	42	40
GO:0098732	macromolecule deacylation	27.3585	1.31E-12	2.69E-11	42	40
GO:0009888	tissue development	27.3313	1.35E-12	2.77E-11	725	411
GO:0070828	heterochromatin organization	27.3092	1.38E-12	2.82E-11	77	64

GO:0042826	histone deacetylase binding	27.2645	1.44E-12	2.95E-11	139	102
GO:0016579	protein deubiquitination	27.2252	1.50E-12	3.07E-11	96	76
GO:0072665	protein localization to vacuole	27.214	1.52E-12	3.10E-11	74	62
GO:1904356	regulation of telomere maintenance via telomere lengthening	27.1936	1.55E-12	3.16E-11	59	52
GO:0071806	protein transmembrane transport	27.1936	1.55E-12	3.16E-11	59	52
GO:0031345	negative regulation of cell projection organization	27.1686	1.59E-12	3.23E-11	218	147
GO:0044774	mitotic DNA integrity checkpoint signaling	26.9279	2.02E-12	4.11E-11	82	67
GO:0051492	regulation of stress fiber assembly	26.8469	2.19E-12	4.44E-11	98	77
GO:0101005	deubiquitinase activity	26.8469	2.19E-12	4.44E-11	98	77
GO:0008135	translation factor activity, RNA binding	26.8232	2.24E-12	4.55E-11	90	72
GO:0009299	mRNA transcription	26.8113	2.27E-12	4.60E-11	52	47
GO:0032872	regulation of stress-activated MAPK cascade	26.7805	2.34E-12	4.74E-11	199	136
GO:0007163	establishment or maintenance of cell polarity	26.7782	2.35E-12	4.74E-11	169	119
GO:0010811	positive regulation of cell-substrate adhesion	26.7425	2.43E-12	4.91E-11	138	101
GO:0055082	intracellular chemical homeostasis	26.7173	2.49E-12	5.03E-11	541	317
GO:0043405	regulation of MAP kinase activity	26.7141	2.50E-12	5.04E-11	192	132
GO:0019318	hexose metabolic process	26.6265	2.73E-12	5.49E-11	164	116
GO:0046605	regulation of centrosome cycle	26.6145	2.76E-12	5.56E-11	55	49
GO:0000725	recombinational repair	26.5956	2.82E-12	5.66E-11	113	86
GO:0070849	response to epidermal growth factor	26.5803	2.86E-12	5.74E-11	41	39
GO:0030218	erythrocyte differentiation	26.5614	2.91E-12	5.84E-11	73	61
GO:1901653	cellular response to peptide	26.5241	3.02E-12	6.06E-11	187	129
GO:0090630	activation of GTPase activity	26.4876	3.14E-12	6.28E-11	100	78
GO:0010634	positive regulation of epithelial cell migration	26.4829	3.15E-12	6.30E-11	159	113
GO:0003724	RNA helicase activity	26.4815	3.16E-12	6.31E-11	70	59
GO:1902750	negative regulation of cell cycle G2/M phase transition	26.4288	3.33E-12	6.64E-11	67	57
GO:0051893	regulation of focal adhesion assembly	26.4083	3.40E-12	6.76E-11	64	55
GO:0090109	regulation of cell-substrate junction assembly	26.4083	3.40E-12	6.76E-11	64	55
GO:0003684	damaged DNA binding	26.4083	3.40E-12	6.76E-11	64	55
GO:0007007	inner mitochondrial membrane organization	26.3956	3.44E-12	6.82E-11	32	32
GO:0033045	regulation of sister chromatid segregation	26.3915	3.45E-12	6.84E-11	105	81
GO:0035303	regulation of dephosphorylation	26.3335	3.66E-12	7.25E-11	125	93
GO:0051015	actin filament binding	26.2934	3.81E-12	7.54E-11	216	145
GO:0016407	acetyltransferase activity	26.1458	4.42E-12	8.71E-11	102	79
GO:2001236	regulation of extrinsic apoptotic signaling pathway	26.139	4.45E-12	8.76E-11	170	119
GO:0090066	regulation of anatomical structure size	26.1048	4.60E-12	9.06E-11	461	275
GO:1904263	positive regulation of TORC1 signaling	26.0843	4.70E-12	9.24E-11	51	46
GO:2000781	positive regulation of double-strand break repair	26.0241	4.99E-12	9.80E-11	86	69

GO:0031124	mRNA 3'-end processing	26.009	5.06E-12	9.94E-11	44	41
GO:0008186	ATP-dependent activity, acting on RNA	25.9115	5.58E-12	1.09E-10	72	60
GO:1902275	regulation of chromatin organization	25.9016	5.64E-12	1.10E-10	54	48
GO:0009416	response to light stimulus	25.8927	5.69E-12	1.11E-10	322	202
GO:0000993	RNA polymerase II complex binding	25.844	5.97E-12	1.17E-10	36	35
GO:0032092	positive regulation of protein binding	25.8202	6.12E-12	1.19E-10	104	80
GO:0000724	double-strand break repair via homologous recombination	25.7719	6.42E-12	1.25E-10	109	83
GO:0008333	endosome to lysosome transport	25.7308	6.69E-12	1.30E-10	63	54
GO:0042789	mRNA transcription by RNA polymerase II	25.6208	7.46E-12	1.45E-10	47	43
GO:0099022	vesicle tethering	25.57	7.85E-12	1.53E-10	31	31
GO:0010822	positive regulation of mitochondrion organization	25.5208	8.25E-12	1.60E-10	77	63
GO:0016055	Wnt signaling pathway	25.4614	8.75E-12	1.70E-10	267	172
GO:0030522	intracellular receptor signaling pathway	25.2956	1.03E-11	2.01E-10	140	101
GO:0098657	import into cell	25.2528	1.08E-11	2.09E-10	613	351
GO:0017069	snRNA binding	25.2522	1.08E-11	2.09E-10	43	40
GO:0008088	axo-dendritic transport	25.2273	1.11E-11	2.14E-10	82	66
GO:0010952	positive regulation of peptidase activity	25.2245	1.11E-11	2.14E-10	191	130
GO:0097191	extrinsic apoptotic signaling pathway	25.224	1.11E-11	2.14E-10	118	88
GO:0042542	response to hydrogen peroxide	25.2226	1.11E-11	2.15E-10	90	71
GO:1990928	response to amino acid starvation	25.191	1.15E-11	2.21E-10	53	47
GO:0007266	Rho protein signal transduction	25.1674	1.17E-11	2.26E-10	68	57
GO:0006986	response to unfolded protein	25.0559	1.31E-11	2.52E-10	62	53
GO:1990841	promoter-specific chromatin binding	25.0525	1.32E-11	2.53E-10	79	64
GO:1900024	regulation of substrate adhesion-dependent cell spreading	25.0523	1.32E-11	2.53E-10	59	51
GO:1900076	regulation of cellular response to insulin stimulus	25.0103	1.37E-11	2.63E-10	87	69
GO:0032231	regulation of actin filament bundle assembly	24.9403	1.47E-11	2.82E-10	115	86
GO:1902017	regulation of cilium assembly	24.8916	1.55E-11	2.96E-10	76	62
GO:1903312	negative regulation of mRNA metabolic process	24.8548	1.61E-11	3.07E-10	92	72
GO:0050769	positive regulation of neurogenesis	24.7956	1.70E-11	3.25E-10	319	199
GO:0042273	ribosomal large subunit biogenesis	24.7443	1.79E-11	3.41E-10	30	30
GO:0009725	response to hormone	24.6777	1.92E-11	3.64E-10	526	306
GO:0031175	neuron projection development	24.6777	1.92E-11	3.64E-10	526	306
GO:0006893	Golgi to plasma membrane transport	24.6366	2.00E-11	3.78E-10	49	44
GO:0009055	electron transfer activity	24.6366	2.00E-11	3.78E-10	49	44
GO:0008408	3'-5' exonuclease activity	24.6366	2.00E-11	3.78E-10	49	44
GO:0051851	modulation by host of symbiont process	24.6142	2.04E-11	3.86E-10	81	65
GO:0051087	protein-folding chaperone binding	24.5454	2.19E-11	4.13E-10	162	113
GO:0004860	protein kinase inhibitor activity	24.5144	2.26E-11	4.26E-10	67	56

GO:0032101	regulation of response to external stimulus	24.494	2.30E-11	4.35E-10	1124	601
GO:0034212	peptide N-acetyltransferase activity	24.4827	2.33E-11	4.39E-10	52	46
GO:0061014	positive regulation of mRNA catabolic process	24.4341	2.45E-11	4.60E-10	64	54
GO:0010972	negative regulation of G2/M transition of mitotic cell cycle	24.4341	2.45E-11	4.60E-10	64	54
GO:0003727	single-stranded RNA binding	24.4287	2.46E-11	4.62E-10	99	76
GO:0031113	regulation of microtubule polymerization	24.3986	2.53E-11	4.76E-10	55	48
GO:0017015	regulation of transforming growth factor beta receptor signalin	24.2879	2.83E-11	5.31E-10	138	99
GO:0008170	N-methyltransferase activity	24.2693	2.88E-11	5.40E-10	91	71
GO:0004843	cysteine-type deubiquitinase activity	24.2693	2.88E-11	5.40E-10	91	71
GO:0050807	regulation of synapse organization	24.2551	2.93E-11	5.48E-10	309	193
GO:0071364	cellular response to epidermal growth factor stimulus	24.2534	2.93E-11	5.48E-10	38	36
GO:0000387	spliceosomal snRNP assembly	24.2484	2.94E-11	5.49E-10	34	33
GO:0140597	protein carrier chaperone	24.2484	2.94E-11	5.49E-10	34	33
GO:0051059	NF-kappaB binding	24.2484	2.94E-11	5.49E-10	34	33
